# Secondary Metabolites with Antimicrobial Activities from *Chamaecyparis obtusa* var. *formosana*

**DOI:** 10.3390/molecules27020429

**Published:** 2022-01-10

**Authors:** Ming-Der Wu, Ming-Jen Cheng, Jih-Jung Chen, Nanthaphong Khamthong, Wen-Wei Lin, Yueh-Hsiung Kuo

**Affiliations:** 1 Bioresource Collection and Research Center (BCRC), Food Industry Research and Development Institute (FIRDI), Hsinchu 300, Taiwan; wmd@firdi.org.tw (M.-D.W.); chengfirdi@gmail.com (M.-J.C.); 2 Department of Pharmacy, School of Pharmaceutical Sciences, National Yang Ming Chiao Tung University (NYCU), Taipei 112, Taiwan; jjungchen@nycu.edu.tw; 3 Department of Medical Research, China Medical University Hospital, Taichung 404, Taiwan; 4 College of Oriental Medicine, Rangsit University, Pathum Thani 12000, Thailand; nanthaphong.k@rsu.ac.th; 5 Department of Chemistry, National Taiwan University, Taipei 106, Taiwan; yang341203@gmail.com; 6 Department of Biotechnology, Asia University, Taichung 413, Taiwan; 7 Department of Chinese Pharmaceutical Sciences and Chinese Medicine Resources, College of Pharmacy, China Medical University, Taichung 404, Taiwan; 8 Chinese Medicine Research Center, China Medical University, Taichung 404, Taiwan

**Keywords:** *Chamaecyparis obtusa* var. *formosana*, *Cupressaceae*, novel skeleton, dimer, diterpenoid, traditional herbal medicine, antimicrobial activities

## Abstract

Seven new compounds, including one dimer novel skeleton, chamaecyformosanin A (**1**); three diterpenes, chamaecyformosanins B–D (**2**–**4**); one sesquiterpene, chamaecyformosanin E (**5**); and two monoterpenes, chamaecyformosanins F and G (**6** and **7**) were isolated from the methanol extract of the bark of *Chamaecyparis obtusa* var. *formosana*. Their structures were established by the mean of spectroscopic analysis and the comparison of NMR data with those of known analogues. Their structures were elucidated on the basis of physicochemical evidence, in-depth NMR spectroscopic analysis, and high-resolution mass spectrometry. Furthermore, the isolated compounds were subjected to an evaluation of their antimicrobial activity. Metabolites **1**, **3**, and **4** present antibacterial activities. It is worth mentioning that the chemical composition of the bark of *C*. *obtusa* var. *formosana* has never been studied in the past. This is the first time the barks from *C*. *obtusa* var. *formosana* were studied and two new skeleton compounds, **1** and **7**, were obtained.

## 1. Introduction

The global distribution of plants of the genus *Chamaecyparis* includes six species and one variety, and they are only distributed in Taiwan [1], Japan, and the United States, except for one species (*C*. *formosensis*) and one variety in Taiwan (*C*. *obtusa* var. *formosana*); there are two Japanese species (*C*. *obtusa* and *C*. *pisifera*) [2,3] and three American species (*C*. *lawsoniana*, *C*. *nootkatensis*, and *C*. *thyoides*) [4]. The Taiwan cypress was collected by Kawakami Takiya and Mori Uzumaki in June 1906 from the Yushan mountain, and was handed over to Hayada Bunzo for publication in 1908. There are many studies in the literature on the composition of plants of the genus Hinoki, and the composition of this genus mainly contains essential oils and lignans [5,6,7,8].

The research object of this paper is Taiwan’s unique cypress family Hinoki (*Chamaecyparis obtusa* var. *formosana*), one of the five largest trees in Taiwan. Taiwan cypress (*Chamaecyparis obtusa* var. *formosana*), also known as yellow juniper or thick crust, is a specialty of Taiwan in the cloud and fog belt at an altitude of 1500 to 2500 m, and most of it is distributed in the area north of the central part, namely MaKau Ecological National Park. It is the most concentrated place in Taiwan. The wood is pungent, aromatic, and highly resistant to decay and insects [5,6,7,8]. The wood is also light, soft, flexible, easy to dry, and less warped. The shrinkage is very small and easy to split. It is easy to plan and process. It has a good nailing performance, good coating, and gluing properties with very high economic value. More importantly, it has high corrosion resistance and ant resistance, so it is regarded as the finest wood.

Nature contains abundant elements of pharmaceutical raw materials. Today, when the pharmaceutical industry is so developed, most drugs are still found in nature or artificially modified products that imitate nature. Although this subject has been developed for a long time, the continuous advancement of analytical instruments and technology today allows us to continuously make new discoveries in the analysis of natural substances [9,10,11]. It also allows us to organize natural objects more accurately and systematically.

**Figure 1:**
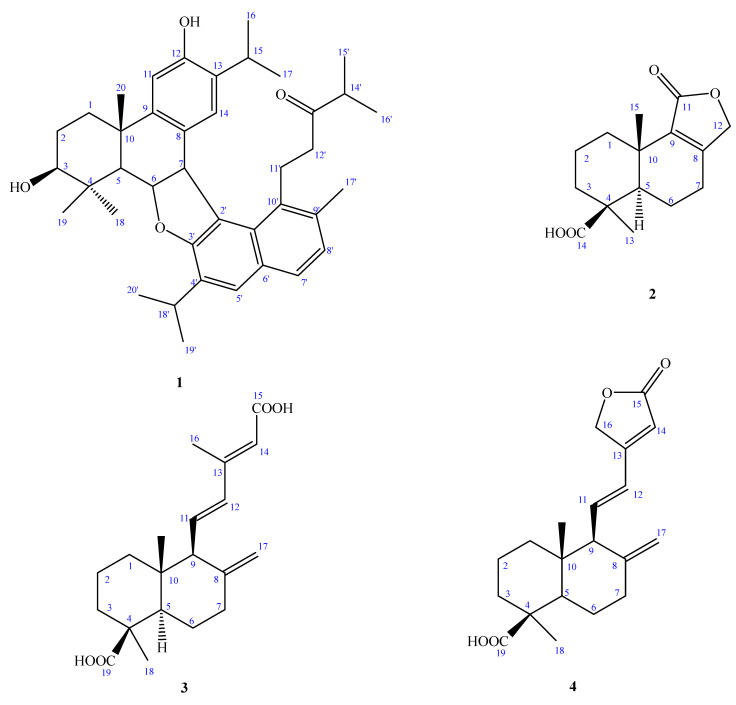

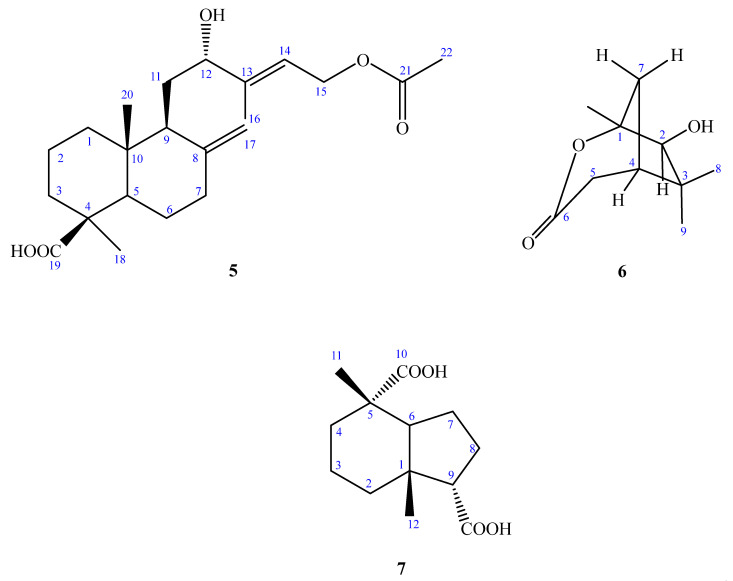
Compounds **1–7**, isolated from *Chamaecyparis obtusa* var. *formosana*.

We have previously investigated the chemical components of the heartwood of this plant and found various monoterpenes, sesquiterpenes, diterpenes, and lignans [1,2,3,4]. The chemical constituents and biological properties from the stem barks of this plant have never been demonstrated. Recently, about 1500 species of Formosan plants have been screened for antimicrobial activity and the bark of *C. obtusa* var. *formosana* was shown to be one of the active species [12]. The MeOH extract of the bark of *C. obtusa* var. *formosana* was suspended in H_2_O and partitioned between H_2_O and EtOAc. The EtOAc-soluble portion was subjected to repeated silica gel column chromatography and semipreparative normal phase-HPLC to afford seven compounds, namely, chamaecyformosanins A–G (**1**–**7**) (Figure 1). The isolation and structural elucidation of these compounds and an assessment of their in vitro antimicrobial activities are described herein.

## 2. Results and Discussion

### 2.1. Structure Elucidation of Compounds

**Figure 2:**
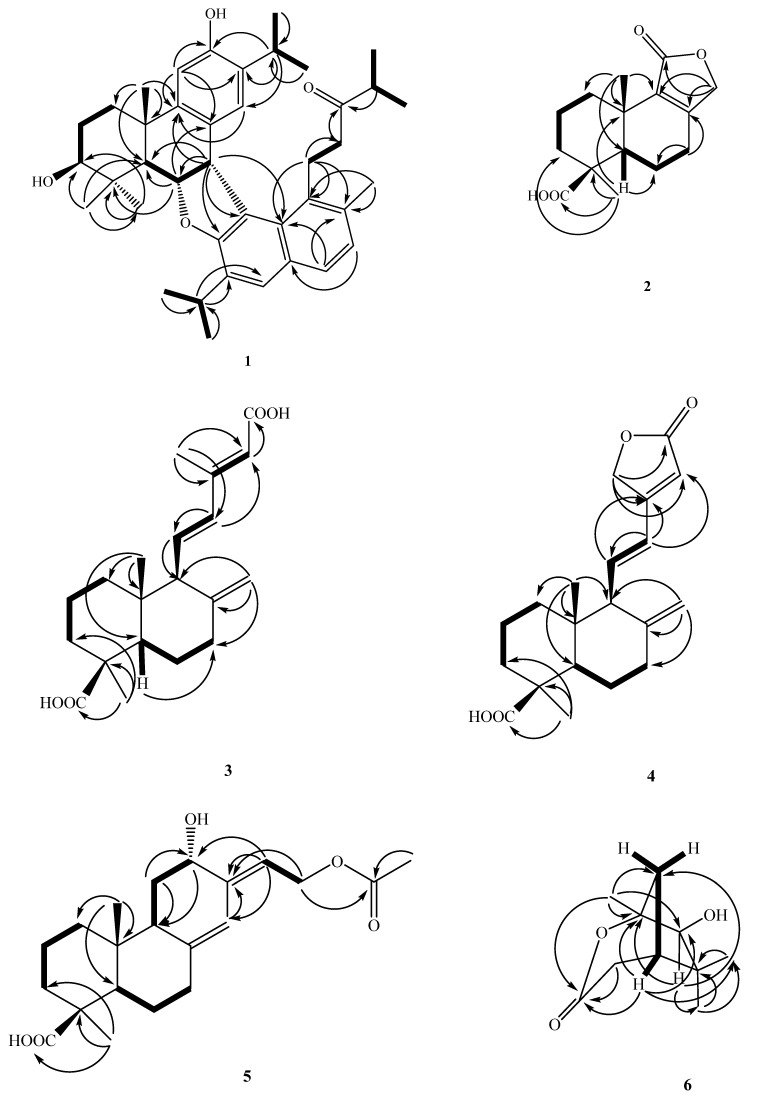

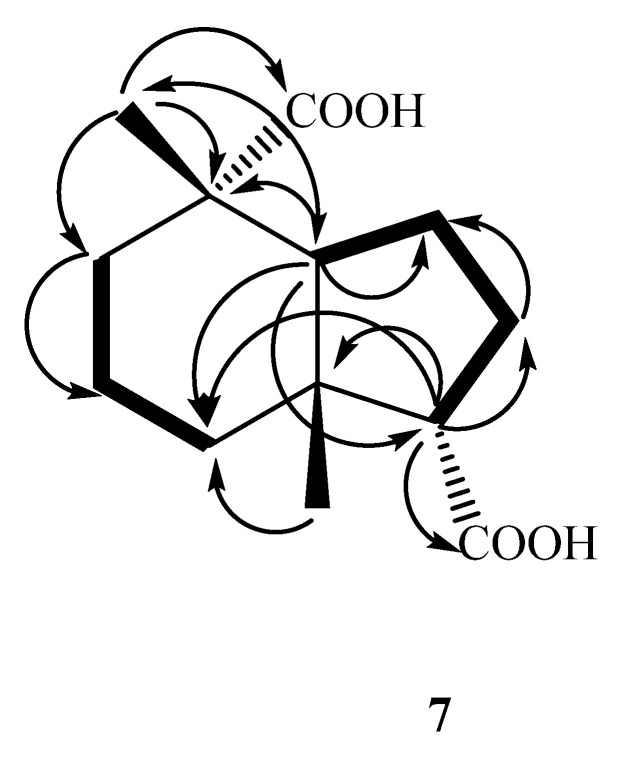
Key COSY (▬) and HMBC (→) correlations of **1–7**.

Compound **1** was isolated as yellowish oil with a positive optical rotation [α ${]}_{D}^{30}$ = + 200.8 (*c* 0.48, CHCl_3_) and UV λ_max_ at 218, 246, 294, and 339 nm, revealing the presence of the conjugated system. The EI-MS of **1** (see in Supplementary Materials) showed a molecular ion peak at *m/z* 596 [M]^+^, and the molecular formula C_40_H_52_O_4_ of **1** was resolved using HR-EI-MS. The IR (KBr) spectrum of **1** showed absorption bands at 3401, 1621, and 1504 cm^−1^ ascribable to hydroxyl and aromatic groups. From the ^1^^3^C-NMR and EI-MS spectrometry analysis, it was found that compound **1** has forty carbons, and its unsaturation is 15; the ^1^H-NMR signals at δ_H_ 2.87 (1H, sept, *J* = 6.9 Hz, H-15), 0.84 (3H, d, *J* = 6.9 Hz, H-16), and 0.67 (3H, d, *J* = 6.9 Hz, H-17) show an isopropyl attached to the benzene ring, and δ_H_ 6.48 (1H, s, H-14) and 6.91 (3H, s, H-11) show the *para*-position on the benzene ring. After examining the ^1^^3^C-NMR spectrum appearing in δ_C_ 151.5 (C-12), 147.0 (C-9), 131.2 (C-13), 127.6 (C-8), 125.7 (C-14), and 109.3 (C-11), the above signals can be determined as benzene moiety. In addition, based on the signals of the three methyl groups of δ_H_ 1.28 (3H, s, CH_3_-20), 1.26 (3H, s, CH_3_-18), and 1.14 (3H, s, CH_3_-19), the above characteristics are inferred to be of an abietane-type skeleton [5]. Comparing the ^1^^3^C-NMR of the compound **1** with 4α-hydroxyferrugiol [13], it was found that the signal is quite close. The difference between them is only that C-3 is connected to a hydroxyl group in **1**, which results in a large difference between C-2, C-3, and C-5, and the ^1^^3^C-NMR signal of C-6 and C-7 is quite different from the literature value. Therefore, it can be inferred that it is possible to use this position to connect with another compound. The ^1^H-NMR spectrum exhibited signals for another benzene ring at δ_H_ 7.58 (1H, d, *J* = 6.8 Hz, H-7′), 7.49 (1H, s, H-5′), and 7.13 (1H, d, *J* = 6.8 Hz, H-8′), and isopropyl groups on a benzene ring at δ_H_ 3.09 (1H, sep, *J* = 6.5 Hz, H-18′), 1.27 (3H, d, *J* = 6.5 Hz, H-20′), and 1.25 (3H, d, *J* = 6.5 Hz, H-19′), while δ_H_ 2.49 (1H, sep, *J* = 6.9 Hz, H-14′), 1.01 (3H, d, *J* = 6.9 Hz, H-16′), and 0.99 (3H, d, *J* = 6.9 Hz, H-15′) are displayed as another isopropyl group. Its ^13^C-NMR signals at δ_C_ 157.3 (C-3′), 134.0 (C-9′), 132.3 (C-10′), 132.0 (C-4′), 130.2 (C-1′), 129.8 (C-6′), 127.4 (C-7′), 126.6 (C-5′), 126.2 (C-8′), and 118.5 (C-2′) can be determined as a naphthalene ring. The signal of δc 214.1 (s) is inferred to be a carbonyl group. Comparing the ^1^H/^13^C-NMR spectra of compound **1** with the known, 1-(7-hydroxy-2,6-dimethyl-1-naphthyl)-4-methyl-3-pentanone [14], they were found to be quite similar. The only difference between the twos was that the C-4′ was changed from a methyl group to an isopropyl group. Therefore, it is inferred that this compound is formed by the dimers of two compounds together. Further using the 2D-NMR HMBC technique, it can be seen that δ_H_ 5.21 (1H, t, *J* = 7.6 Hz) is correlated with δ_C_ 127.6 (C-8), 52.8 (C-5), 46.2 (C-7), and 39.8 (C-4), and δ_H_ 4.74 (1H, d, *J* = 7.6 Hz) is related to δ_C_ 157.3 (s), 147.0 (s), 130.2 (s), 127.6 (s), 118.5 (s), and 84.0 (d), inferring that C-6 is related to the oxygen atom of C-3′ and they are connected to each other, C-7 is connected to C-2′ by C–C linkage, and displayed from the COSY signals (Figure 2), δ_H_ 5.21 (t, 7.6 Hz, 1H) and δ_H_ 4.74 (1H, d, *J* = 7.6 Hz) and 1.74 (1H, d, *J* = 7.6 Hz) are correlated; the correlations between δ_H_ 1.26 (s, 3H), 1.14 (s, 3H), and δ_C_ 78.7 (d, C-3) were disclosed in the HMBC plot (Figure 2). From the IR absorption spectrum, it was found that there is a hydroxyl (-OH) signal at 3401 cm^−1^, so it was determined that δ_H_ 5.21 (1H, t, *J* = 7.6 Hz) is H-6, 4.74 ((1H, t, *J* = 7.6 Hz) is H-7, 1.74 (1H, t, *J* = 7.6 Hz) is H-5, and 3.39 (1H, dd, *J* = 8.9, 6.9 Hz) is H-3 with a hydroxyl group. Therefore, it was determined that compound **1** is composed of two individual ones using C–C bonds and an oxygen atom to form a dimer.

**Figure 3:**
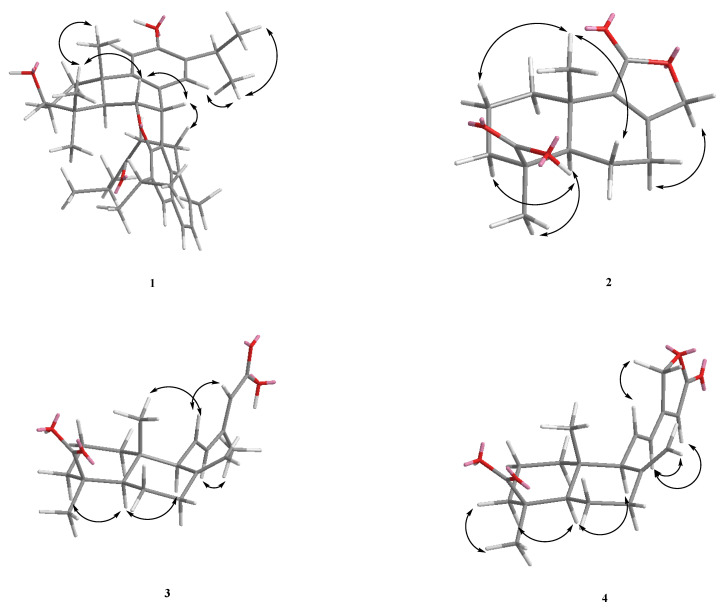

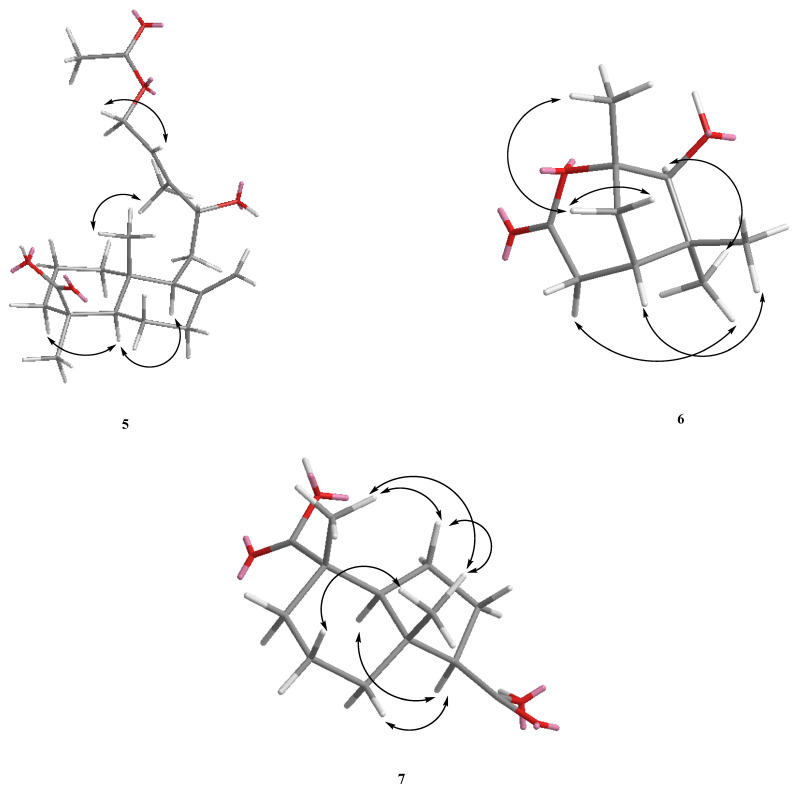
Major NOESY (↔) contacts of **1**–**7**.

From the NOESY spectrum, H-20 is correlated to H-6 and H-19. Therefore, it can be confirmed that C-20 and C-19 are in the axial position, and the ^1^H-NMR signal of H-18 has a lower magnetic field, so it was inferred that an oxygen atom attached to C-6 should be located in the equatorial position. Therefore, H-18 is affected by the lone pair electron on the oxygen atom and it is displaced to the lower magnetic field, that is, H-6 is located in the axial position. H-18 is correlated to H-3 and H-5 but not related to H-20. It can be determined that the CH_3_-19 on C-4 is located on the axial, and the hydroxyl (-OH) on C-3 occupies the equatorial position (Figure 3).

The presence of NOE cross-peaks between H-6 and H-7 clearly established the β-equatorial orientation of the H-7. The C–C linkage bond between C-7 and C-2′ was assigned the α-axial orientation, since the naphthalene ring and diterpenoid exhibited vertical orientations, causing the chemical shift of H-14, H-15, H-16, and H-17 with a higher magnetic field than usual. Then, other 2D-NMR spectra were used to determine the compound **1** as chamaecyformosanin A.

Chamaecyformosanin B (**2**) was obtained as yellowish oil with dextrorotatory optical activity. Its molecular formula was established as C_15_H_20_O_4_ by the EI-MS (M^+^ at *m/z* 264) and HR-EI-MS. The UV maxima at 215 and 259 nm suggested the presence of a conjugated γ-lactone functionality. The absorption bands for COOH (2500~3400 cm^−^^1^), and conjugated carbonyl (1739 cm^−^^1^) groups were observed in its IR spectrum. The signals of the ^13^C-NMR spectrum at δ_C_ 159.5 (C-8) and 134.4 (C-9) can be inferred to contain a group of four substituted double bonds and have a signal of δ_C_ 172.3 (C-11), which is inferred to be a conjugated γ-lactone, and the signal from δ_C_ 183.4 (C-14) is a non-conjugated carboxyl group. The ^13^C-NMR spectrum indicated that there were 15 carbon atoms, the unsaturation of **2** is estimated to be 6 by the molecular formula. After deducting one conjugate γ-lactone and a group of carboxyl groups, the remaining unsaturation is 2, and then the two methyl groups located at δ_H_ 1.28 (s) and 1.04 (s) are deducted. The remaining compound is 10 carbons, so it was deduced that compound **2** is a drimane-type sesquiterpene with two six-membered rings.

Observed from the HMBC spectrum, δ_H_ 4.54 and 4.59 (each 1H, d, *J* = 17.0 Hz, H-12) are correlated to δ_C_ 172.3 (C-11), 159.5 (C-8), 134.4 (C-9), and 25.6 (C-7), so it is confirmed to be a conjugated γ-lactone. Then, according to the correlation between δ_H_ 1.28 (s, 3H, H-13) and δ_C_ 183.4 (C-14), 53.2 (C-5), 37.5 (C-3), and 35.4 (C-4) and δ_H_ 1.04 (3H, s, H-15) and δ_C_ 134.4 (C-9), 53.2 (C-5), 34.6 (C-1), and 43.4 (C-10) being related, it can be determined that δ_H_ 1.28 (3H, s) and carborylic acid (COOH) are connected to C-4. The NOE correlations of H-13/H-3, H-13/H-5, and no NOE contacts of H-15/H-13, 5 confirmed a *trans*-fused decalin ring in **2**. The orientation of C-15 being positioned at the axial and C-13 being located on the equatorial were confirmed. The double bond with oxygen was in position C-11 and not on C-12 based on the NOE correlations of H-7/H-12 and aided by the HMBC correlation between δ_H_ 4.54, 4.59 (H-12) and δ_C_ 25.6 (C-7). The ^1^H- and ^13^C-NMR (Table 1), HMBC (Figure 2), COSY (Figure 2), and NOESY (Figure 3) were compatible with the structure of **2** as in Figure 1, named chamaecyformosanin B.

Compound **3** was obtained as a yellowish oil, and the molecular formula was determined to be C_20_H_28_O_4_ by HR-EI-MS (*m*/*z* 332.1960 [M]^+^ (calcd for C_20_H_28_O_4_, 332.1982)). The IR spectrum showed the presence of COOH (2500~3400 cm^−1^) and conjugated carbonyl (1693 cm^−1^) groups. The ^1^H (Table 2) and ^13^C NMR (Table 1) data of **3** were very similar to those of 15-oxolabda-8(17),11(*E*),13(*E*)-triene-19-oic acid [15], except that a carboxylic acid group (δ_c_ 172.6 (conjugated COOH)) at C-15 in **3** replaced the aldehyde group (δ_H_ 10.07 (1H, d, *J* = 8.0 Hz, CHO); δ_c_ 191.0 (CHO)) of 15-oxolabda-8(17),11(*E*),13(*E*)-triene-19-oic acid. The relative configuration of **3** was assigned by the NOESY spectrum, which showed a correlation between H-18 (δ_H_ 1.25) and H-3 (δ_H_ 2.18(1H, br d, *J* = 12.3 Hz)/5 (δ_H_ 1.33 (1H, dd, *J* = 12.0, 2.5 Hz), suggesting that both CH_3_-4 and H-5 were was on the α configuration. The *E*-configuration at C-11 was confirmed by the NOESY correlation between H-14 and H-12, between H-16 and H-11, and between H-11 and H-20. This was supported by the HMBC correlation between H-14 (δ_H_ 5.71) and C-15 (δ_C_ 172.6) (Figure 2). The full assignment of ^1^H and ^13^C NMR resonances was supported by ^1^H–^1^H COSY (Figure 2), DEPT, HSQC, NOESY, and HMBC (Figure 2) spectral analyses. Thus, the structure of **3** was established as shown in Figure 1, and named chamaecyformosanin C.

Compound **4** was isolated as yellowish oil. Its molecular formula, C_20_H_2__8_O_4_, was determined on the basis of the HR-EI-MS at *m/z* 330.1877 [M]^+^ (calcd for 330.1871) and was supported by the ^1^H, ^13^C, and DEPT NMR data. The IR absorption bands of **4** revealed the presence of COOH (2500~3400 cm^−1^), COOH (1696 cm^−1^), and conjugated γ-lactone (1778 cm^−1^) functions. The ^1^H- (Table 2) and ^13^C-NMR (Table 1) data of **4** were similar to those of 12-hydroxylabda-8(17),13-dien-18-oic acid-15,16-olide [16], except that one *E*-configuration double at C-11-12 (δ_H_ 6.37 (1H, d, *J* = 16.0 Hz, H-12), 6.13 (1H, dd, *J* = 16.0, 10.6 Hz, H-11); δ_C_ 139.2 (C-11), 124.2 (C-12)) in **4** replaced the CH_2_-CH(OH)- group [δ_H_ 1.75/2.20 (2H, m, H-11), 4.60 (1H, d, *J* = 9.6 Hz, H-12); δ_C_ 32.1 (C-11), 71.5 (C-12)] at the C-11-12 position of 12-hydroxylabda-8(17),13-dien-18-oic acid-15,16-olide [16]. The double bond with oxygen was in position C-15 and not in C-16 according to the NOE correlations of H-11/H-16. The full assignment of ^1^H and ^13^C NMR resonances was supported by ^1^H–^1^H COSY (Figure 2), DEPT, HSQC, NOESY (Figure 3), and HMBC (Figure 2) spectral analyses. Thus, the structure of **4** was established as shown in Figure 3, and named chamaecyformosanin D.

Compound **5** was obtained as yellowish oil. Its molecular formula C_22_H_34_O_5_ was deduced from a molecular ion peak at *m/z* 378.2022 [M]^+^ (calcd 378.2406) in the HREI mass spectrum. The presence of COOH (2500~3400 cm^−1^), 1696 (COOH), and ester carbonyl (1681 cm^−1^) groups were evident from the IR spectrum. The ^1^H (Table 2) and ^13^C-NMR (Table 1) data of **5** were very similar to those of 15-acetoxylabda-8(17),13(*E*)-dien-19-oic acid [17], except that an oxymethine group (δ_H_ 4.12 (1H, dd, *J* = 9.0, 5.5 Hz; δ_C_ 77.3)) at C-12 in **5** replaced the methylene group (δ_H_ 0.98/1.81(2H, m; δ_C_ 38.4)) of 15-acetoxylabda-8(17),13(*E*)-dien-19-oic acid (acetylisocupressic acid) [17]. In order to determine the absolute configuration of C-12, after comparing the ^1^H- and ^13^C NMR spectra of (12*S*)-hydroxylabda-8(17),13(Z)-dien-12,19-dioic acid [18], it was found to be quite close to compound **5**. The only difference between the two structures was the orientation of the double bond at the C-13 position and the acetyl group at C-15. According to the literature [10], if the steric orientation of the compound (12*S*) of the labdane structure is determined to be *S*, the steric orientation makes the hydroxyl group (-OH) closer to the terminal double bond protons, causing one of the ^1^H-NMR chemical shift values (δ) of C-17 to move to about δ_H_ 4.70 (br s) in the low field, and the signal of one of the protons at the C-17 position of compound **5**, located at δ_H_ 4.66 (br s), is very close to (12*S*)-hydroxylabda-8(17),13(*Z*)-dien-12,19-dioic acid, so it is speculated that the C-12 stereo orientation of compound **5** is *S*.

The full assignment of ^1^H and ^13^C NMR resonances was supported by ^1^H–^1^H COSY (Figure 2), DEPT, HSQC, NOESY, and HMBC (Figure 2) spectral analyses. Thus, the structure of **5** was established as shown in Figure 1, and named chamaecyformosanin E.

Compound **6** was obtained as an optically active yellowish gum with [α ${]}_{D}^{30}$ = −14.0 (*c* 0.008, CHCl_3_). The molecular formula was determined as C_1__0_H_1__6_NO_3_, with three indices of hydrogen deficiency on the basis of the [M]^+^ peak at *m/z* 184.1095 in its HR-EI-MS. Bands at 3430 and 1726 cm^−1^ in the IR spectrum revealed the presence of a hydroxy group and ester moiety, respectively. The above inference can be confirmed by examining the signals of the ^13^C-NMR spectrum, δ_C_ 84.6 (d) and 170.3 (s). The unsaturation degree of **6** should be 3 calculated by the molecular formula, and the remaining unsaturation degree is 2 after subtracting one ester group. From the ^1^H-NMR spectrum, there are three Me at δ_H_ 1.42 (3H, s, CH_3_-10), 1.15 (3H, s, CH_3_-9), and 1.04 (3H, s, CH_3_-8). The signal next to the carbonyl group is at δ_H_ 2.47 (1H, dd, *J* = 18.8, 4.8 Hz, CH_2_-5α) and 2.71 (1H, dd, *J* = 18.8, 2.4 Hz, CH_2_-5β), so it was deduced that compound **6** is a monoterpenoid fencane-type structure, and then 2D HMBC was further used to confirm this. The signal at δ_H_ 1.42 (CH_3_-10) is correlated to δ_C_ 170.3 (C-6), 91.9 (C-1), 84.6 (C-2), and 38.0 (C-7), so it can be confirmed that ester is connected to C-1 instead of δ_C_ 84.6 (C-2). Because if an ester is connected to δ_C_ 84.6 (C-2), δ_H_ 1.42 (CH_3_-10) will be too far away (^5^*J*) and has no HMBC correlation with δ_C_ 170.3 (C-6). Based on the above ^1^H- and ^13^C-NMR, COSY (Figure 2), NOESY (Figure 3), DEPT, HSQC, and HMBC (Figure 2) experiments, the structure of **6** was elucidated as (2*R*,4*R*)-2-hydroxy-1,3,3-trimethyl-2-oxabicyclo[3.2.1]octan-6-one and named as chamaecyformosanin F.

Compound **7** was obtained as an optically active colorless gum ([α ${]}_{D}^{30}$ + 42.4 (*c* 0.13, CHCl_3_)) and showed a molecular ion M^+^ peak at *m/z* 240.1367 for C_13_H_20_O_4_, corresponding to four indices of hydrogen deficiency (IHD). The IR spectrum of **7** displayed absorptions for COOH groups (2500~3300 cm^−1^) and COOH (1697 cm^−1^). The ^1^H- and ^13^C-NMR (Table 2), COSY (Figure 2), HMBC (Figure 2), and NOESY data (Figure 3) established the structure of **7** as (1b,4b,5a,10a)-1,4-epoxymuurolan-5-ol. The ^13^C-NMR spectrum exhibited 13 signals for two Me, five CH_2_, and two CH groups, and for four quaternary C atoms (including two COOH (δ_C_ 184.3 (C-10) and 180.2 (C-13)). After deducting the two groups of carboxyl groups, the remaining unsaturation is 2, plus the two methyl groups of δ_H_ 1.21 (Me-11) and 0.62 (Me-12), leaving compound **7** with nine carbons. Further use was made of 2D-HMBC to find the following correlations (Figure 2): from CH_3_-11 (δ_H_ 1.21 (3H, s)) to C-10 (δ_C_ 184.3), C-6 (δ_C_ 57.9), C-5 (δ_C_ 43.9), and C-4 (δ_C_ 37.1); from CH_3_-12 (δ_H_ 0.62 (3H, s)) to C-6 (δ_C_ 57.9), C-9 (δ_C_ 55.9), C-1 (δ_C_ 44.5), and C-2 (δ_C_ 39.3); from H-6 (δ_H_ 1.40 (1H, dd, *J* = 12.8, 7.3 Hz)) to C-10 (δ_C_ 184.3), C-9 (δ_C_ 55.9), C-5 (δ_C_ 43.9), C-2 (δ_C_ 39.3), C-11 (δ_C_ 27.9), and C-7 (δ_C_ 22.6); and from H-9 (δ_H_ 2.38 (1H, t, *J* = 9.2 Hz)) to C-13 (δ_C_ 180.2), C-1 (δ_C_ 44.5), C-2 (δ_C_ 39.3), C-8 (δ_C_ 21.7), and C-12 (δ_C_ 12.4). These along with the following COSY correlations (Figure 2): H–3 (δ_H_ 1.82/1.52 (each 1H, m))/H–4 (δ_H_ 0.91 (1H, td, *J* = 13.5, 4.3 Hz, H-4_equ_)/2.17 (1H, m, H-4_ax_))/H-2 (δ_H_ 1.20 (1H, m, H-2_ax_)/2.10(1H, d, *J* = 13.0 Hz, H-2_equ_)) and H-6 (δ_H_ 1.40 (1H, dd, *J* = 12.8, 7.3 Hz, H-6_ax_))/H-7 (δ_H_ 1.83/2.18 (each 1H, m))/H-8 (δ_H_ 1.72/2.09 (each 1H, m))/H-9 (δ_H_ 2.38 (1H, t, *J* = 9.2 Hz, H-9_ax_)), established that the planar structure of **7** can be inferred to be composed of a six-membered and a five-membered ring. Therefore, it was determined that compound **7** is a new skeleton. From the above results, it can be determined that δ_H_ 1.21 (s, 3H) and δ_H_ 0.62 (s, 3H) are undoubtedly located on C-4 and C-8, and δ_C_ 184.3/180.2 were assigned to C-4/C-1. The assignments were further verified by significant correlations of H–11 (δ_H_ 1.21 (3H, s)/H-12 (δ_H_ 0.62 (3H, s); H-6 (δ_H_ 1.40)/H-3 (δ_H_ 1.82); CH_3_–12 (δ_H_ 0.62 (3H, s)/H-7 (δ_H_ 1.83/2.18); as well as H-9 (δ_H_ 2.383)/H-6 (δ_H_ 1.40)/H-2 (δ_H_ 1.20/2.10) in the NOESY experiments (Figure 3) and further supported the positions of each substituent in **7**. Therefore, it was confirmed that the compound **7** is (1,4,8)-octahydro-4,8-dimethyl-1*H*-indene-1,4-dicarboxylic acid, and it was named as chamaecyformosanin G.

### 2.2. Biological Studies

The antimicrobial activities of the five isolates from the barks of *C. obtusa* var. *formosana* were tested against bacteria such as *Staphylococcus aureus* subsp. *aureus* (BCRC 10451), *Bacillus subtilis* subsp. *subtilis* (BCRC-10255), *Pseudomonas aeruginosa* (BCRC-11633), *Klebsiella pneumoniae* subsp. *pneumoniae* (BCRC-16082), and *Escherichia coli* (BCRC-11634), as well as the following fungi: *Aspergillus niger* (BCRC-31512), *Penicillium italicum* (BCRC-30567), *Candida albicans* (BCRC-21538), and *Saccharomyces cerevisiae* (BCRC-20822). The antimicrobial data are shown in Table 3 and clinically used antimicrobial agents, tetracycline (for bactericidal) and ketoconazole (for fungicidal), were used as positive controls. Due to the small quantity of **6** and **7**, the five isolates (**1**–**5**) in sufficient amounts had their antimicrobial activities evaluated. Our results indicated that metabolites **1**, **3**, and **4** present antimicrobial activities, which were absent in **2** and **5**. From the results of the antimicrobial tests, the following conclusions can be drawn regarding these isolates: (a) the novel skeleton (**1**) showed moderate antibacterial and weak antifungal activities compared to the standard drugs tetracycline and ketoconazole. It indicated the inhibition zones of 21~24 mm against *Staphylococcus aureus* subsp. *aureus* (BCRC 10451), *Bacillus subtilis* subsp. *subtilis* (BCRC-10255), *Escherichia coli* (BCRC-11634), and *Pseudomonas aeruginosa* (BCRC-11633), and showed moderate to weak antifungal activities with inhibition zones of 12, 13, 20, and 19 mm against *Aspergillus niger* (BCRC-31512) *Penicillium italicum* (BCRC-30567), *Candida albicans* (BCRC-21538), and *Saccharomyces cerevisiae* (BCRC-20822), respectively, compared to the ketoconazole. (b) The diterpenes, chamaecyformosanins C and D (**3** and **4**), exhibited strong antibacterial and weak antifungal activities against all tested strains. (c) Chamaecyformosanin C (**3**) indicated the strong inhibition zones of 30, 29, 28, and 29 mm against *Staphylococcus aureus* subsp. *aureus* (BCRC 10451), *Bacillus subtilis* subsp. *subtilis* (BCRC-10255), *Escherichia coli* (BCRC-11634), and *Pseudomonas aeruginosa* (BCRC-11633), and showed weak antifungal activities with inhibition zones of 17, 20, 19, and 17 mm against *Aspergillus niger* (BCRC-31512) *Penicillium italicum* (BCRC-30567), *Candida albicans* (BCRC-21538), and *Saccharomyces cerevisiae* (BCRC-20822), respectively. (d) Chamaecyformosanin D (**4**) indicated the strong inhibition zones of 27, 29, 29, and 28 mm against *Staphylococcus aureus* subsp. *aureus* (BCRC 10451), *Bacillus subtilis* subsp. *subtilis* (BCRC-10255), *Escherichia coli* (BCRC-11634), and *Pseudomonas aeruginosa* (BCRC-11633), and also showed weak antifungal activities with inhibition zones of 19, 16, 19, and 17 mm against *Aspergillus niger* (BCRC-31512) *Penicillium italicum* (BCRC-30567), *Candida albicans* (BCRC-21538), and *Saccharomyces cerevisiae* (BCRC-20822) compared to the standard drug ketoconazole.

## 3. Materials and Methods

### 3.1. General Experimental Procedures

TLC: silica gel 60 F_254_ precoated plates (Merck, Darmstadt, Germany). Column chromatography (CC): silica gel 60 (70–230 or 230–400 mesh, Merck) and Spherical C18 100A Reversed Phase Silica Gel (RP-18) (particle size: 20–40 μm) (SiliCycle, Quebec City, Canada). HPLC: Spherical C18 column (250 mm × 10 mm, 5μm) (Waters, Milford, MA, USA); LDC-Analytical-III apparatus. UV Spectra: Jasco UV-240 spectrophotometer; λ_max_ (log ε) in nm. Optical rotation: Jasco DIP-370 polarimeter; in CHCl_3_. IR Spectra: Perkin-Elmer-2000 FT-IR spectrophotometer; ν in cm^−1^. ^1^H-, ^13^C-, and 2D-NMR spectra: Varian-Mercury-500 and Varian-Unity-Plus-400 spectrometers; *δ* in ppm rel. to Me_4_Si, *J* in Hz. ESI and HRESIMS: Bruker APEX-II mass spectrometer; in *m*/*z*.

### 3.2. Plant Material

The barks of *C*. *obtusa* var. *formosana* were collected from Taichung, Taiwan, in August 1996. The plant was identified by Dr. Shang-Tzen Chang, Professor of the Department of Forestry, National Taiwan University. A voucher specimen (No. Kuo-9) has been deposited in the Herbarium of the Department of Botany of the National Taiwan University, Taipei, Taiwan.

### 3.3. Isolation and Characterization of Secondary Metabolites

The air-dried bark of *C*. *obtusa* var. *formosana* (12.1 kg) was extracted two times with acetone (100 L) at room temperature (every 7 days). The acetone extract was concentrated, and the black residue was suspended in H_2_O (7 L) and then extracted with EtOAc. The EtOAc fraction (709 g) was subjected to CC (silica gel, hexane/EtOAc of increasing polarity (H (100)→H: E (95:5)→H: E(90:10)→H: E (85:15)→H: E (80:20)→H: E (70:30)→H: E (50:50)→H: E (30:70)→E (100); then using EtOAc/MeOH of increasing polarity (E: M (90: 10)→E: M (70: 30)→E: M (50: 50)→M(100)) to give 12 fractions (1–12). Fr. 6 (69.3 g) was subjected to CC (Sephadex LH-20, 1.0 cm × 30.0 cm, MeOH) to give four fractions: Frs. 6-1~6-4. Fr. 6-1 (18.1 g) was purified by semi-preparative HPLC (30% EtOAc/CH_2_Cl_2_ in 8 min→20% acetone/hexane in 12 min→10% MeOH/H_2_O 3 min, flow rate 2.5 mL/min) to yield **1** (5.4 mg). Fr. 6-2 (22.1 g) was purified by semi-preparative HPLC (30% EtOAc/CH_2_Cl_2_ in 8 min→25% acetone/hexane in 12 min→10% MeOH/H_2_O 3 min, flow rate 2.5 mL/min) to yield **2** (24.5 mg)**, 5** (7.9 mg), **6** (1.4 mg), and **7** (0.9 mg), respectively. Fr. 8 (100.4 g) was purified by semi-preparative HPLC (15% EtOAc/CH_2_Cl_2_ in 12 min→10% acetone/hexane in 6 min, flow rate 2.5 mL/min) to yield **3** (14.1 mg) and **4** (6.2 mg).

Chamaecyformosanin A (**1**): yellowish oil; [α ${]}_{D}^{30}$ = +200.8 (*c* 0.48, CHCl_3_); UV (MeOH): 339 (3.7), 294 (4.0), 246 (4.7), 218 (4.7) nm; IR (Neat): 3401(-OH), 1621, 1504 (benzene ring) cm^−1^; ^1^H NMR (500 MHz, CDCl_3_): see Table 2; ^13^C NMR (125 MHz, CDCl_3_): see Table 1; HREIMS *m/z*:596.3861 [M]^+^ (calcd for C_40_H_52_O_4_, 596.3866).

Chamaecyformosanin B (**2**): yellowish oil; [α ${]}_{D}^{30}$ = +56.0 (*c* 0.98, CHCl_3_); UV (MeOH): 259 (3.11), 215 (4.11) nm; IR (Neat): 2500–3400, 1696 (COOH), 1739 (conjugate γ-lactone) cm^−1^; ^1^H NMR (500 MHz, CDCl_3_): see Table 2; ^13^C NMR (125 MHz, CDCl_3_): see Table 1; EIMS (70 eV) *m/z* (%): 264 ([M]^+^, 19), 246 (26), 219 (24), 203 (100), 112 (42); HREIMS *m/z*: 264.1352 [M–H_2_O]^+^ (calcd for C_1__5_H_2__0_O_4_, 264.1362).

Chamaecyformosanin C (**3**): yellowish oil; [α ${]}_{D}^{30}$ = −22.6 (*c* 0.59, CHCl_3_); UV (MeOH): 258 (4.36) nm; IR (Neat): 2500–3400, 1693 (COOH), 1650, 3078, 893 (C = CH_2_), 1632, 1441, 978 cm^−1^; ^1^H NMR (500 MHz, CDCl_3_): see Table 2; ^13^C NMR (125 MHz, CDCl_3_): see Table 1; EIMS (70 eV) *m/z* (%): 332 ([M]^+^, 64), 314 (74), 145 (23), 105 (30); HREIMS *m/z*: 332.1960 [M]^+^ (calcd for C_20_H_28_O_4_, 332.1982).

Chamaecyformosanin D (**4**): yellowish oil; [α ${]}_{D}^{30}$ = –9.4 (*c* 0.26, CHCl_3_); UV (MeOH): 259 (4.21) nm; IR (Neat): 2500–3400, 1696 (COOH), 1778, 1747 (conjugated γ-lactone, C = O), 3085, 1647, 890(C = CH_2_) cm^–^^1^; ^1^H NMR (500 MHz, CDCl_3_): see Table 2; ^13^C NMR (125 MHz, CDCl_3_): see Table 1; EIMS (70 eV) *m/z* (%): 330 ([M]^+^, 41), 312 (9), 285 (12), 121 (98), 55 (100); HREIMS *m/z*: 330.1877 [M]^+^ (calcd for C_20_H_28_O_4_, 330.1871).

Chamaecyformosanin E (**5**): yellowish oil; [α ${]}_{D}^{30}$ = +19.9 (*c* 0.33, CHCl_3_); IR (Neat): 2500~3400 (COOH), 1694 (COOH), 1681 (ester C = O) cm^–^^1^; ^1^H NMR (500 MHz, CDCl_3_): see Table 2; ^13^C NMR (125 MHz, CDCl_3_): see Table 1; EIMS (70 eV) *m/z* (%): 377 ([M-H]^+^, 100), 348 (36), 331 (60), 305 (35), 275(14); HREIMS *m/z*: 378.2022 [M]^+^ (calcd for C_22_H_34_O_5_, 378.2406).

Chamaecyformosanin F (**6**): yellowish gum; [α ${]}_{D}^{30}$ = −14.0 (*c* 0.17, CHCl_3_); IR (Neat): 3430 (OH), 1726 (ester C = O) cm^–^^1^; ^1^H NMR (500 MHz, CDCl_3_): see Table 2; ^13^C NMR (125 MHz, CDCl_3_): see Table 1; EIMS (70 eV) *m/z* (%): 183 ([M-H]^+^, 43); HREIMS *m/z*: 184.1095 [M]^+^ (calcd for C_10_H_16_O_3_, 184.1099).

Chamaecyformosanin G (**7**): colorless gum; [α ${]}_{D}^{30}$ = +42.4 (*c* 0.13, CHCl_3_); IR (Neat): 2500~3300 (COOH), 1697 (COOH) cm^–^^1^; ^1^H NMR (500 MHz, CDCl_3_): see Table 2; ^13^C NMR (125 MHz, CDCl_3_): see Table 1; EIMS (70 eV) *m/z* (%): 239 ([M-H]^+^, 100), 193 (5); HREIMS *m/z*: 240.1367 [M]^+^ (calcd for C_13_H_20_O_4_, 240.1362.2406).

### 3.4. Antimicrobial Activity Assays

The in vitro antimicrobial activities of compounds **1**–**5** was tested against a panel of laboratory control strains belonging to the Bioresource Collection and Research Center (BCRC), Hsinchu, Taiwan. The Gram-positive tested microorganisms were *Staphylococcus aureus* subsp. *aureus* (BCRC 10451), and *Bacillus subtilis* subsp. *subtilis* (BCRC-10255), and the Gram-negative ones were *Pseudomonas aeruginosa* (BCRC-11633), *Klebsiella pneumoniae* subsp. *pneumoniae* (BCRC-16082), and *Escherichia coli* (BCRC-11634), and fungal organisms *Aspergillus niger* (BCRC-31512), *Penicillium italicum* (BCRC-30567), *Candida albicans* (BCRC-21538), and *Saccharomyces cerevisiae* (BCRC-20822).

The disc diffusion method according to the NCCLS [19] was employed for the determination of the antimicrobial activities of the compounds. Briefly, a suspension of the tested microorganisms (0.1 mL of 10^8^ cells per mL) was spread on the solid media plates. The following nutritive media were used: Antibiotic Medium 1 (Difco Laboratories, Detroit, Michigan, USA) for growing Gram-positive and Gram-negative bacteria and Tryptone soy agar (TSA; Torlak, Belgrade) for *Aspergillus niger*, *Penicillium italicum*, *Candida albicans*, and *Saccharomyces cerevisiae*. Nutritive media were prepared according to the instructions of the manufacturer. All agar plates were prepared in 90 mm Petri dishes with 22 mL of agar, giving a final depth of 4 mm. Sterile filter paper disks (8 mm in diameter; Advantec, Tokyo, Japan) were impregnated with 50 μL of the sample solution in dimethyl sulfoxide (DMSO), 1 mg/1 mL of DMSO (all solutions were filter-sterilized using a 0.45 mm membrane filter), and placed on inoculated plates. These plates, after standing at 4 °C for 2 h, were incubated at 37 °C for 24 h for bacteria and at 30 °C for 48 h for the fungi. Standard disks of tetracycline (for bactericidal purposes) and ketoconazole (for fungicidal purposes) were used as positive controls, while the disk imbued with 50 μL of pure DMSO was used as a negative control. The diameters of the inhibition zones were measured in millimeters and by means of a slide caliper. Each test was performed in triplicate and repeated three times.

## 4. Conclusions

*C*. *obtusa* var. *formosana* (also called *Taiwan cypress,* *Taiwan* yellow cedar, and Formosan hinoki) is a variant plant endemic to Taiwan. It grows in the middle and upper part of a hillside or the flat part of a ridge at a high altitude of about 1300–2700 m in the Central Mountain Range. It is also occasionally seen in depressions and highlands. The wood is extremely good for various uses: the wood material is dense and tough, without cracking, and is mainly used for construction, furniture, sleepers, bridges, shipbuilding, sculptures, and decorations. In addition, it has been reported in medicinal practice that essential oils containing hinokitiol and rhodinic acid have an antibiotic effect, and rhodinic acid can treat tuberculosis. This study explored seven new components in the bark of *C*. *obtusa* var. *formosana* that have not been published previously. Compounds **1** and **7** are new skeletons. We also screened sufficient compounds for antimicrobial properties (anti-fungi and bacteria assays), and the results were found that the new skeleton (**1**) has good antibacterial activity, one sesquiterpene (**2**) is inactive, and two diterpenoids (**3** and **4**) have moderate antibacterial activity. The results of this screening show the weak antifungal activity compared to that of ketoconazole. The active substances with a unique structure and antibacterial activity make it an interesting material to be further developed.

## Figures and Tables

**Table 1:** ^13^C-NMR Data for Compounds **1**–**7** (*δ* in ppm, 125 MHz for ^13^C NMR in CDCl_3_).

No	1	2	3	4	5	6	7
1	36.9	34.6	40.9	41.0	39.0	91.9	44.5
2	27.4	18.7	19.6	19.5	19.9	84.6	39.3
3	78.7	37.5	38.0	38.0	37.9	44.5	20.1
4	39.8	35.4	44.1	44.0	44.1	42.0	37.1
5	52.8	53.2	55.6	55.3	56.2	35.7	43.9
6	84.0	19.9	24.9	24.8	26.0	170.3	57.9
7	46.2	25.6	37.2	37.0	38.6	38.0	22.8
8	127.6	159.5	148.9	148.2	148.3	24.6	21.7
9	147.0	134.4	60.6	60.8	52.4	26.4	55.9
10	36.9	43.4	40.0	40.4	40.2	20.3	184.3
11	109.3	172.3	136.2	139.2	28.7		27.9
12	151.5	70.6	136.1	124.2	77.3		12.4
13	131.2	28.7	154.8	161.7	142.4		180.2
14	125.7	183.4	117.5	114.6	121.8		
15	26.3	17.6	172.6	174.0	60.9		
16	22.0		14.3	70.5	10.7		
17	22.2		108.4	108.7	106.9		
18	28.3		28.9	28.9	28.9		
19	20.9		183.9	182.3	183.0		
20	16.2		13.7	13.7	12.9		
1′	130.2						
2′	118.5						
3′	157.3						
4′	132.0						
5′	126.6						
6′	129.8						
7′	127.4						
8′	126.2						
9′	134.0						
10′	132.3						
11′	24.0						
12′	41.1						
13′	214.1						
14′	41.0						
15′	18.4						
16	18.0						
17′	20.6						
18′	28.6						
19′	21.3						
20′	22.6						

**Table 2:** ^1^H-NMR Data for Compounds **1**–**7** in CDCl_3_ (*δ* in ppm, *J* in Hz, 500 MHz in CDCl_3_).

No	1	2	3	4	5	6	7
1	1.78 (m)	1.16 (dd, *J* = 13.3, 4.1)	1.07 (m)	1.09 (m)	0.95 (m)		
	2.17 (m)	2.56 (d, *J* = 13.3)	1.46 (m)	1.44 (m)	1.79 (m)		
2	1.89 (m)	1.53 (m, H-2α)	1.46 (m)	1.76 (m)	1.50 (m)	3.62 (d, *J* = 2.4)	1.20 (m, H-2α(ax))
	1.91 (m)	1.88 (td, *J* = 14.0, 3.6, H-2β)	1.85 (m)	1.83 (m)	1.83 (m)		2.10 (d, J = 13.0, H-2β(equ))
3	3.39 (dd, *J* = 8.9, 6.9)	1.08 (dd, *J* = 14.0, 3.6)	1.05 (m)	1.06(m)	1.03 (m)		1.52 (m)
		2.22 (m)	2.18 (br d, J = 12.3)	2.18(m)	2.13 (m)		1.82 (m)
4						1.95 (td, *J* = 4.8, 1.6)	0.91 (td, J = 13.5, 4.3, H-4α(equ))
							2.17(m, H-4α(ax))
5	1.74 (d, *J* = 7.6)	1.40 (dd, *J* = 12.2, 1.3)	1.33 (dd, *J* = 12.0, 2.5)	1.33 (dd, *J* = 12.5, 2.7)	1.27 (m)	2.47 (dd, *J* = 18.8, 4.8)	
						2.71 (dt, *J* = 18.8, 2.4)	
6	5.21 (t, *J* = 7.6)	1.95 (m)	1.90 (m)	1.89 (m)	1.83 (m)		1.40 (dd, *J* = 12.8, 7.3)
		2.17 (m)	1.98 (m)	1.99 (m)	1.94 (m)		
7	4.74 (d, *J* = 7.6)	2.26 (m)	2.00 (m)	2.01 (m)	1.82 (m)	1.68 (dd, *J* = 12.8, 2.4)	1.83 (m)
		2.37 (m)	2.45 (m)	2.46 (m)	2.38 (m)	2.12 (ddd, *J* = 12.8, 4.8, 2.4)	2.18 (m)
8						1.04 (s)	1.72 (m)
							2.09 (m)
9			2.43 (m)	2.47 (m)	1.42 (m)	1.15 (s)	2.38 (t, *J* = 9.3)
10						1.42 (s)	
11	6.91 (s)		6.21 (dd, *J* = 15.6, 9.8)	6.13 (dd, *J* = 16.0, 10.6)	1.27 (m)		1.21 (s)
					1.68 (m)		
12		4.54 (d, *J* = 17.0)	6.09 (d, *J* = 15.6)	6.37 (d, *J* = 16.0)	4.12 (dd, *J* = 9.0, 5.5)		0.62 (s)
		4.59 (d, *J* = 17.0)					
13		1.28 (s)					
14	6.48 (s)		5.71 (s)	5.85 (*s*)	5.40 (t, *J* = 6.4)		
15	2.87 (sept, *J* = 6.9)	1.04 (s)			4.60 (dd, *J* = 9.0, 6.4)		
16	0.84 (d, *J* = 6.9)		2.29 (s)	4.97 (br s)	1.67 (s)		
17	0.67 (d, *J* = 6.9)		4.41 (br s)		4.66 (br s)		
			4.76 (br s)		4.87 (br s)		
18	1.26 (s)		1.25 (s)		1.21 (s)		
19	1.14 (s)						
20	1.28 (s)		0.76 (s)		0.59 (s)		
21							
22					2.04 (s)		
1′							
2′							
3′							
4′							
5′	7.49 (s)						
6′							
7′	7.58 (d, *J* = 6.8)						
8′	7.13 (d, *J* = 6.8)						
9′							
10′							
11′	3.10 (m),3.33 (m)						
12′	2.64 (m), 2.68 (m)						
13′							
14′	2.49 (sept, *J* = 6.9)						
15′	0.99 (d, *J* = 6.9)						
16′	1.01 (d, *J* = 6.9)						
17′	2.46 (s)						
18′	3.09 (sept, *J* = 6.5)						
19′	1.25 (d, *J* = 6.5)						
20′	1.27 (d, *J* = 6.5)						

**Table 3:** Antimicrobial activities of five sufficient compounds isolated from the bark of *C. obtusa* var. *formosana* (diameter of the zone of growth inhibition, bactericidal or fungicidal zone in mm, including the diameter of the disc, 8 mm).

Test Microorganism	Isolated Compounds					Tetracycline	Ketoconazole
	1	2	3	4	5		
*S. aureus* subsp*. aureus*	24	–	30	27	–	25	–
*B. subtilis* subsp*. subtilis*	25	–	29	29	–	24	–
*P. aeruginosa*	22	–	28	29	–	24	–
*E. coli*	21	–	29	28	–	23	–
*A*. *niger*	12	–	17	19	–	–	32
*P. italicum*	13	–	20	16	–	–	30
*C*. *albicans*	20	–	19	19	–	–	29
*S*. *cerevisiae*	19	–	17	17	–	–	32

Inhibition zone diameter (mm); – = no inhibition zone; positive controls: tetracycline and ketoconazole.

## Data Availability

Not applicable.

## Supplementary Materials

The following supporting information can be downloaded, Figure S1. ^1^H NMR spectrum of **1**, Figure S2. ^13^C NMR spectrum of **1**, Figure S3. ^1^H-^1^H COSY spectrum of **1**, Figure S4. HMBC spectrum of **1**, Figure S5. NOESY spectrum of **1**, Figure S6. HSQC spectrum of **1**, Figure S7. EI-MS spectrum of **1**, Figure S8. ^1^H NMR spectrum of **2**, Figure S9. ^13^C NMR spectrum of **2**, Figure S10. ^1^H-^1^H COSY spectrum of **2**, Figure S11. HMBC spectrum of **2**, Figure S12. NOESY spectrum of **2**, Figure S13. HSQC spectrum of **2**, Figure S14. EI-MS spectrum of **2**, Figure S15. ^1^H NMR spectrum of **3**, Figure S16. ^13^C NMR spectrum of **3**, Figure S17. ^1^H-^1^H COSY spectrum of **3**, Figure S18. HMBC spectrum of **3**, Figure S19. NOESY spectrum of **3**, Figure S20. HSQC spectrum of **3**, Figure S21. EI-MS spectrum of **3**, Figure S22. ^1^H NMR spectrum of **4**, Figure S23. ^13^C NMR spectrum of **4**, Figure S24. ^1^H-^1^H COSY spectrum of **4**, Figure S25. HMBC spectrum of **4**, Figure S26. NOESY spectrum of **4**, Figure S27. HSQC spectrum of **4**, Figure S28. EI-MS spectrum of **4**, Figure S29. ^1^H NMR spectrum of **5**, Figure S30. ^13^C NMR spectrum of **5**, Figure S31. ^1^H-^1^H COSY spectrum of **5**, Figure S32. HMBC spectrum of **5**, Figure S33. NOESY spectrum of **5**, Figure S34. HSQC spectrum of **5**, Figure S35. EI-MS spectrum of **5**, Figure S36. ^1^H NMR spectrum of **6**, Figure S37. ^13^C NMR/DEPT spectra of **6**, Figure S38. ^1^H-^1^H COSY spectrum of **6**, Figure S39. HMBC spectrum of **6**, Figure S40. NOESY spectrum of **6**, Figure S41. HSQC spectrum of **6**, Figure S42. EI-MS spectrum of **6**, Figure S43. ^1^H NMR spectrum of **7**, Figure S44. ^13^C NMR/DEPT spectra of **7**, Figure S45. ^1^H-^1^H COSY spectrum of **7**, Figure S46. HMBC spectrum of **7**, Figure S47. NOESY spectrum of **7**, Figure S48. HSQC spectrum of **7**, Figure S49. EI-MS spectrum of **7**.

## Sample Availability

Samples of the compounds are not available from the authors.
