# Correction: Wu et al. Secondary Metabolites with Antimicrobial Activities from *Chamaecyparis* *obtusa* var. *formosana*. *Molecules* 2022, 27, 429

**DOI:** 10.3390/molecules27186048

**Published:** 2022-09-16

**Authors:** Ming-Der Wu, Ming-Jen Cheng, Jih-Jung Chen, Nanthaphong Khamthong, Wen-Wei Lin, Yueh-Hsiung Kuo

**Affiliations:** 1 Bioresource Collection and Research Center (BCRC), Food Industry Research and Development Institute (FIRDI), Hsinchu 300, Taiwan; wmd@firdi.org.tw (M.-D.W.); chengfirdi@gmail.com (M.-J.C.); 2 Department of Pharmacy, School of Pharmaceutical Sciences, National Yang Ming Chiao Tung University (NYCU), Taipei 112, Taiwan; jjungchen@nycu.edu.tw; 3 Department of Medical Research, China Medical University Hospital, Taichung 404, Taiwan; 4 College of Oriental Medicine, Rangsit University, Pathum Thani 12000, Thailand; nanthaphong.k@rsu.ac.th; 5 Department of Chemistry, National Taiwan University, Taipei 106, Taiwan; yang341203@gmail.com; 6 Department of Biotechnology, Asia University, Taichung 413, Taiwan; 7 Department of Chinese Pharmaceutical Sciences and Chinese Medicine Resources, College of Pharmacy, China Medical University, Taichung 404, Taiwan; 8 Chinese Medicine Research Center, China Medical University, Taichung 404, Taiwan

The authors wish to make the following changes to their paper [1].

**Author List**

Original:

Ming-Der Wu ^1^, Ming-Jen Cheng ^1,^*, Jih-Jung Chen ^2,3,^*, Nanthaphong Khamthong ^4^, Wen-Wei Lin ^5^ and Yueh-Hsiung Kuo ^3,^^5,6,7^

^1^ Bioresource Collection and Research Center (BCRC), Food Industry Research and Development Institute (FIRDI), Hsinchu 300, Taiwan 

^2^ Department of Pharmacy, School of Pharmaceutical Sciences, National Yang Ming Chiao Tung University (NYCU), Taipei 112, Taiwan

^3^ Department of Medical Research, China Medical University Hospital, Taichung 404, Taiwan; yhkuo800@gmail.com

^4^ College of Oriental Medicine, Rangsit University, Pathum Thani 12000, Thailand; nanthaphong.k@rsu.ac.th

^5^ Department of Chemistry, National Taiwan University, Taipei 106, Taiwan; yang341203@gmail.com

^6^ Department of Biotechnology, Asia University, Taichung 413, Taiwan

^7^ Department of Chinese Pharmaceutical Sciences and Chinese Medicine Resources, College of Pharmacy, China Medical University, Taichung 404, Taiwan

* Correspondence: chengfirdi@gmail.com (M.-J.C.); jjungchen@nycu.edu.tw (J.-J.C.)

To be replaced with:

Ming-Der Wu ^1,^^†^, Ming-Jen Cheng ^1^, Jih-Jung Chen ^2,3^, Nanthaphong Khamthong ^4^, Wen-Wei Lin ^5,^^†^ and Yueh-Hsiung Kuo ^6,7,8,^*

^1^ Bioresource Collection and Research Center (BCRC), Food Industry Research and Development Institute (FIRDI), Hsinchu 300, Taiwan; wmd@firdi.org.tw (M.-D.W.); chengfirdi@gmail.com (M.-J.C.)

^2^ Department of Pharmacy, School of Pharmaceutical Sciences, National Yang Ming Chiao Tung University (NYCU), Taipei 112, Taiwan; jjungchen@nycu.edu.tw

^3^ Department of Medical Research, China Medical University Hospital, Taichung 404, Taiwan

^4^ College of Oriental Medicine, Rangsit University, Pathum Thani 12000, Thailand; nanthaphong.k@rsu.ac.th

^5^ Department of Chemistry, National Taiwan University, Taipei 106, Taiwan; yang341203@gmail.com

^6^ Department of Biotechnology, Asia University, Taichung 413, Taiwan

^7^ Department of Chinese Pharmaceutical Sciences and Chinese Medicine Resources, College of Pharmacy, China Medical University, Taichung 404, Taiwan

^8^ Chinese Medicine Research Center, China Medical University, Taichung 404, Taiwan

* Correspondence: kuoyh@mail.cmu.edu.tw

† These authors contributed equally to this work.

**Author Contribution**

Original:

Y.-H.K., J.-J.C. and M.-J.C. designed the research; N.K., M.-D.W.,W.-W.L. and M.-J.C. performed the research; N.K. conducted biological assays, J.-J.C., Y.-H.K. and M.-J.C. helped with structure elucidation; M.-J.C. organized the data and wrote the paper. All authors have read and agreed to the published version of the manuscript.

To be replaced with:

Y.-H.K. is responsible for the main research framework; Y.-H.K. and M.-J.C. designed the research; W.-W.L. performed the chemical research; N.K. and M.-D.W. conducted antimicrobial assays, J.-J.C., Y.-H.K. and M.-J.C. helped with structure elucidation; Y.-H.K. and M.-J.C. organized the data and M.-J.C. wrote the paper. All authors have read and agreed to the published version of the manuscript.

The authors apologize for any inconvenience caused and state that the scientific conclusions are unaffected. This correction was approved by the Academic Editor. The original publication has also been updated.
