# Supplementary material for: Secondary Metabolites with Antimicrobial Activities from Chamaecyparis obtusa var. formosana
Source: Molecules. 2022 Jan 10;27(2):429. doi: 10.3390/molecules27020429 (PMC8779593; doi:10.3390/molecules27020429)
Supplement: Supplementary file 1 [file molecules-27-00429-s001.zip › molecules-1415538-supplementary.pdf]

Supplementary File

Secondary Metabolites with Antimicrobial Activities from *Chamaecyparis obtusa* var. *formosana*

Ming-Der Wu[1], Ming-Jen Cheng[1,*], Jih-Jung Chen[2,3,*], Nanthaphong Khamthong[4], Wen-Wei Lin[5] and Yueh-Hsiung Kuo[3,5,6,7]

[1] Bioresource Collection and Research Center (BCRC), Food Industry Research and Development Institute (FIRDI), Hsinchu 300, Taiwan
[2] Department of Pharmacy, School of Pharmaceutical Sciences, National Yang Ming Chiao Tung University (NYCU), Taipei 112, Taiwan
[3] Department of Medical Research, China Medical University Hospital, Taichung 404, Taiwan
[4] College of Oriental Medicine, Rangsit University, Pathum Thani 12000, Thailand
[5] Department of Chemistry, National Taiwan University, Taipei 106, Taiwan
[6] Department of Biotechnology, Asia University, Taichung 413, Taiwan
[7] Department of Chinese Pharmaceutical Sciences and Chinese Medicine Resources, College of Pharmacy, China Medical University, Taichung 404, Taiwan

* Correspondences: chengfirdi@gmail.com (M.-J.C.); jjungchen@nycu.edu.tw (J.-J.C.)

Contents

Figure S1. ^{1}H NMR spectrum of **1** 5

Figure S2. ^{13}C NMR spectrum of **1** 5

Figure S3. ^{1}H-^{1}H COSY spectrum of **1** 6

Figure S4. HMBC spectrum of **1** 6

Figure S5. NOESY spectrum of **1** 7

Figure S6. HSQC spectrum of **1** 7

Figure S7. EI-MS spectrum of **1** 8

Figure S8. ^{1}H NMR spectrum of **2** 9

Figure S9. ^{13}C NMR spectrum of **2** 9

Figure S10. ^{1}H-^{1}H COSY spectrum of **2** 10

Figure S11. HMBC spectrum of **2** 10

Figure S12. NOESY spectrum of **2** 11

Figure S13. HSQC spectrum of **2** 11

Figure S14. EI-MS spectrum of **2** 12

Figure S15. ^{1}H NMR spectrum of **3** 13

Figure S16. ^{13}C NMR spectrum of **3** 13

Figure S17. ^{1}H-^{1}H COSY spectrum of **3** 14

Figure S18. HMBC spectrum of **3** 14

Figure S19. NOESY spectrum of **3** 15

Figure S20. HSQC spectrum of **3** 15

Figure S21. EI-MS spectrum of **3** 16

Figure S22. ^{1}H NMR spectrum of **4** 17

Figure S23. ^{13}C NMR spectrum of **4** 17

Figure S24. ^{1}H-^{1}H COSY spectrum of **4** 18

Figure S25. HMBC spectrum of **4** 18

Figure S26. NOESY spectrum of **4** 19

Figure S27. HSQC spectrum of **4** 19

Figure S28. EI-MS spectrum of **4** 20

Figure S29. ^{1}H NMR spectrum of **5** 21

Figure S30. ^{13}C NMR spectrum of **5** 21

Figure S31. ^{1}H-^{1}H COSY spectrum of **5** 22

Figure S32. HMBC spectrum of **5** 22

Figure S33. NOESY spectrum of **5** 23

Figure S34. HSQC spectrum of **5** 23

Figure S35. EI-MS spectrum of **5** 24

Figure S36. ^{1}H NMR spectrum of **6** 25

Figure S37. ^{13}C NMR/DEPT spectra of **6** 25

Figure S38. ^{1}H-^{1}H COSY spectrum of **6** 26

Figure S39. HMBC spectrum of **6** 26

Figure S40. NOESY spectrum of **6** 27

Figure S41. HSQC spectrum of **6** 27

Figure S42. EI-MS spectrum of **6** 28

Figure S43. ^{1}H NMR spectrum of **7** 29

Figure S44. ^{13}C NMR/DEPT spectra of **7** 29

Figure S45. ^{1}H-^{1}H COSY spectrum of **7** 30

Figure S46. HMBC spectrum of **7** 30

Figure S47. NOESY spectrum of **7** 31
Figure S48. HSQC spectrum of **7** 31
Figure S49. EI-MS spectrum of **7** 32

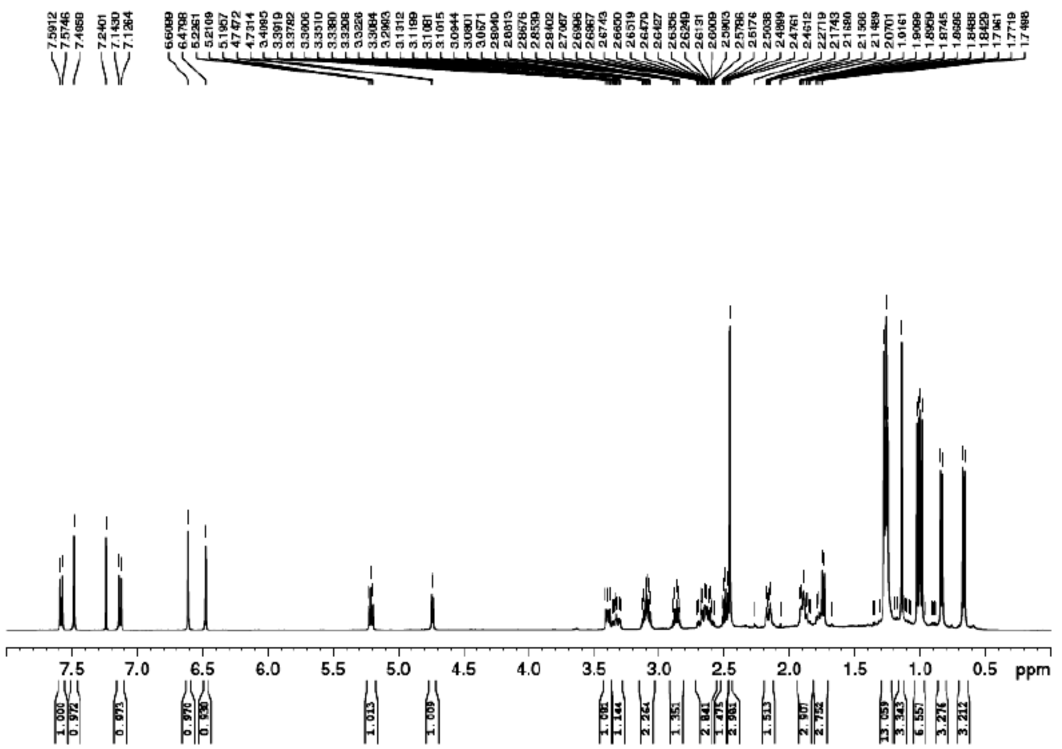

Figure S1. ^{1}H NMR spectrum of **1**

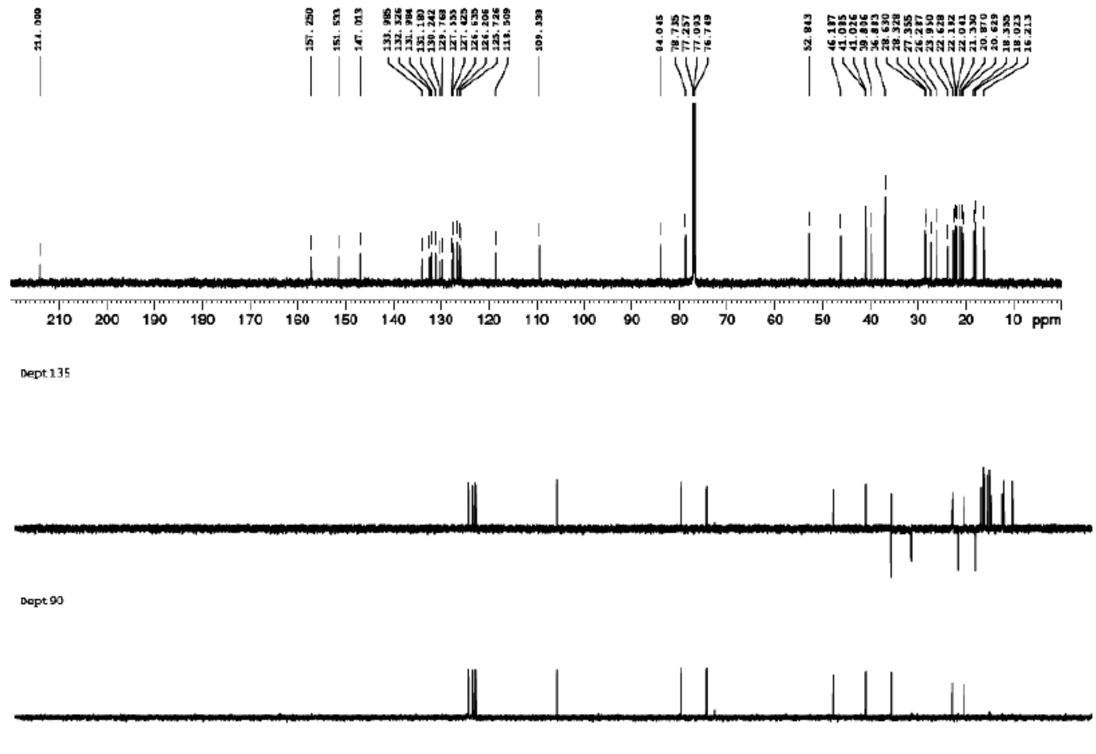

Figure S2. ^{13}C NMR spectrum of **1**

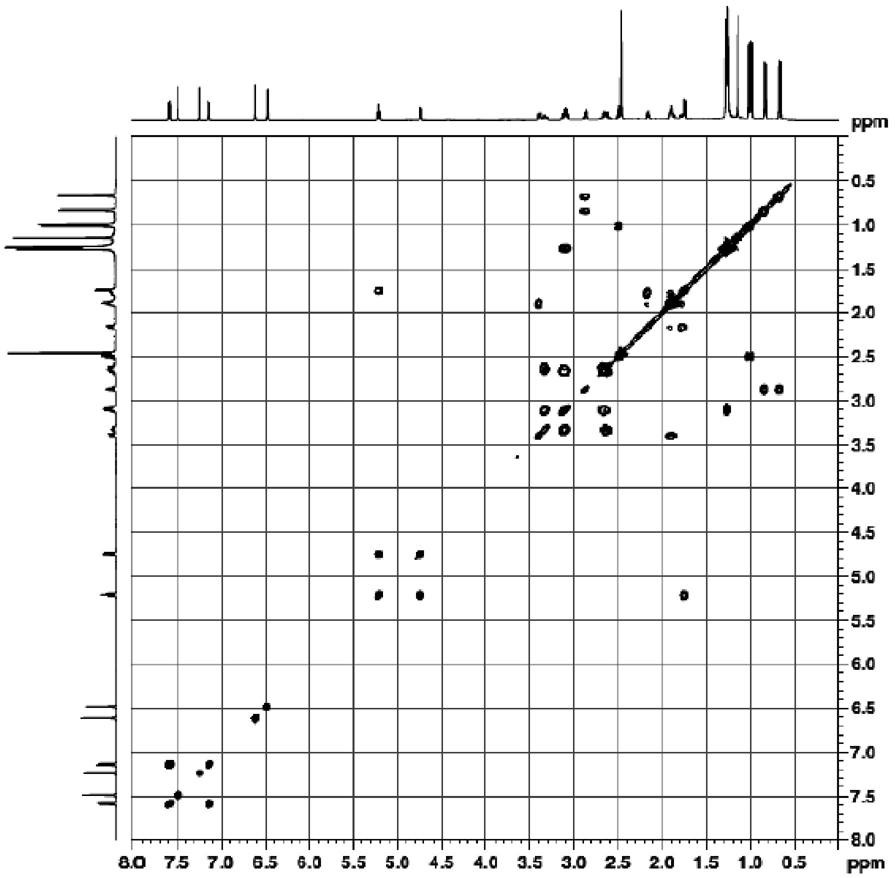

Figure S3. COSY spectrum of **1**

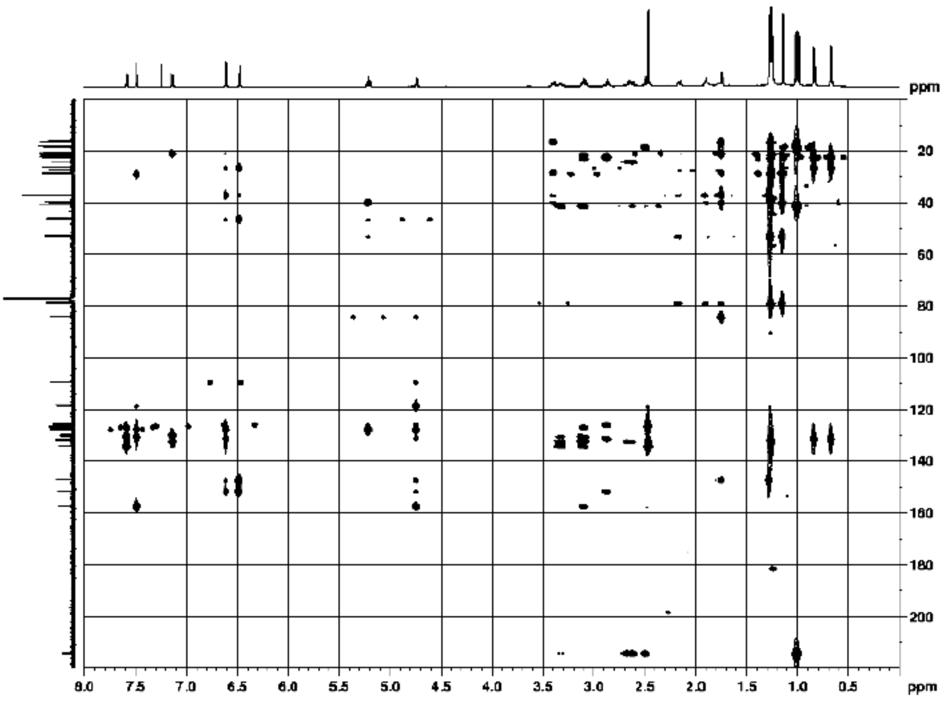

Figure S4. HMBC spectrum of **1**

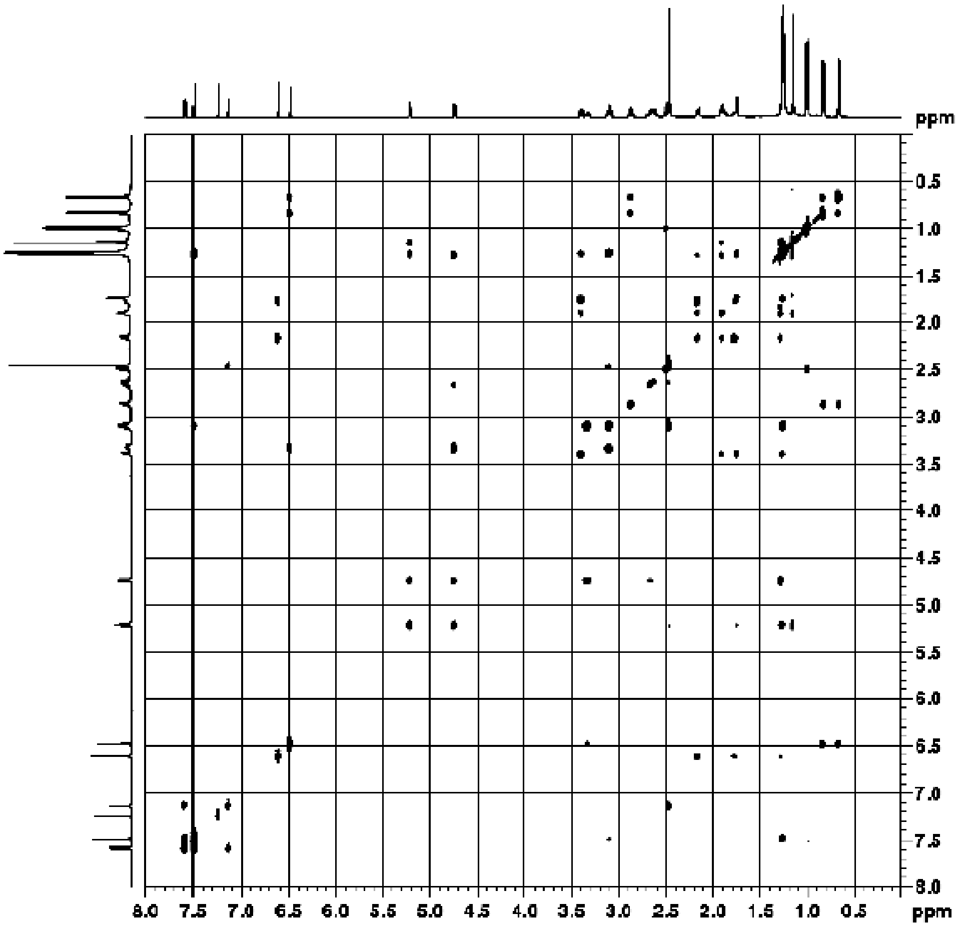

Figure S5. NOESY spectrum of **1**

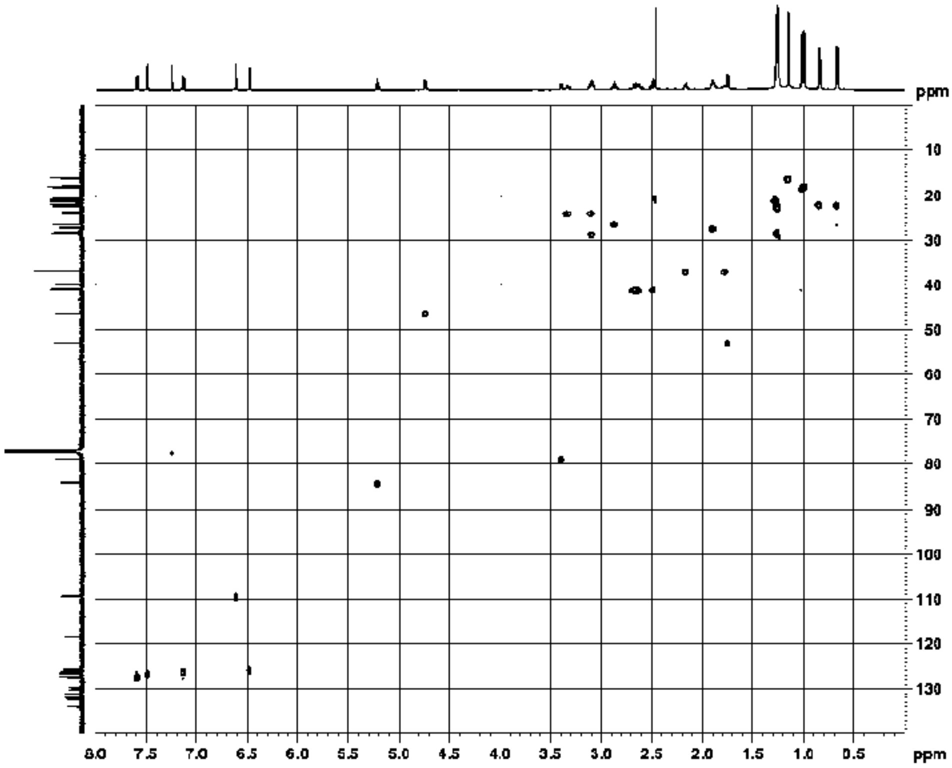

Figure S6. HSQC spectrum of **1**

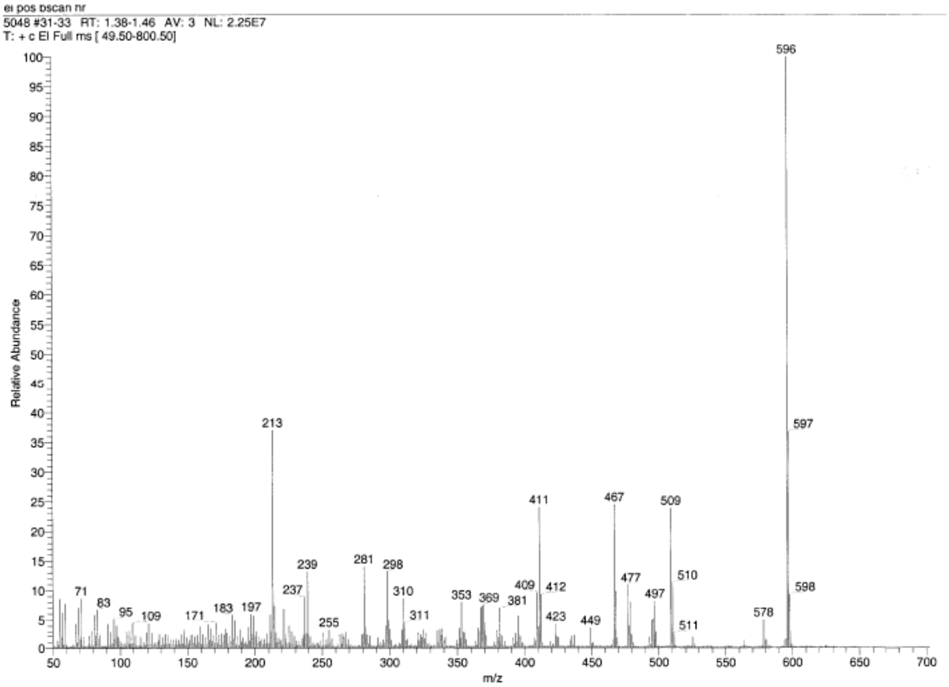

Figure S7. EIMS spectrum of **1**

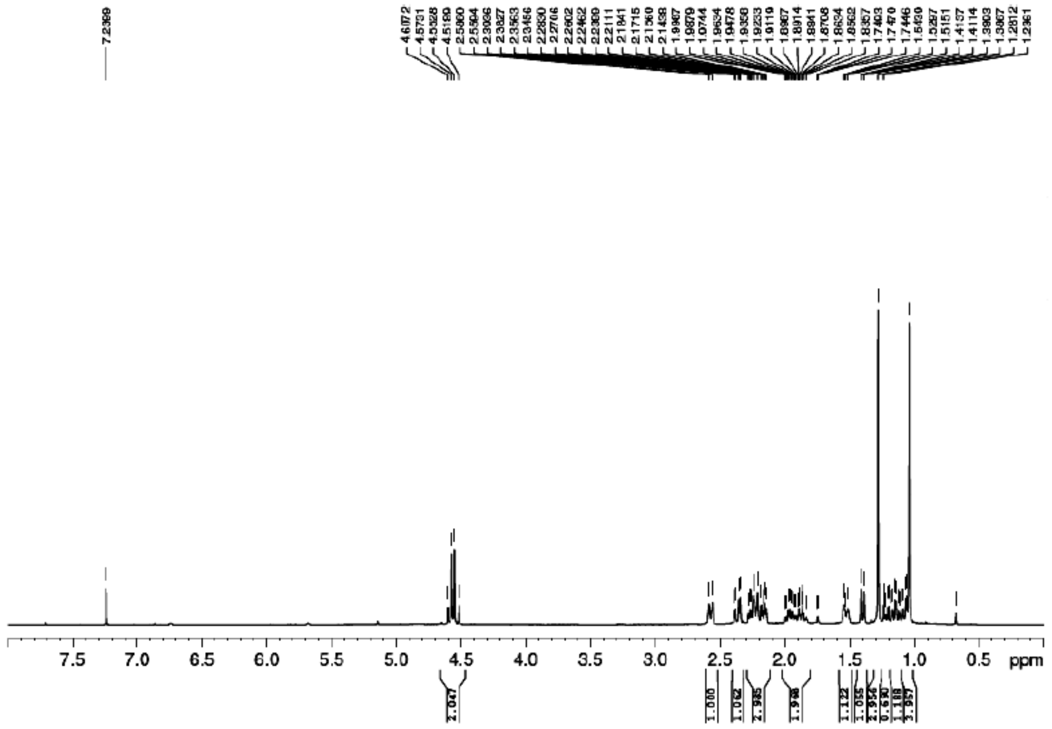

Figure S8. ^{1}H NMR spectrum of **2**

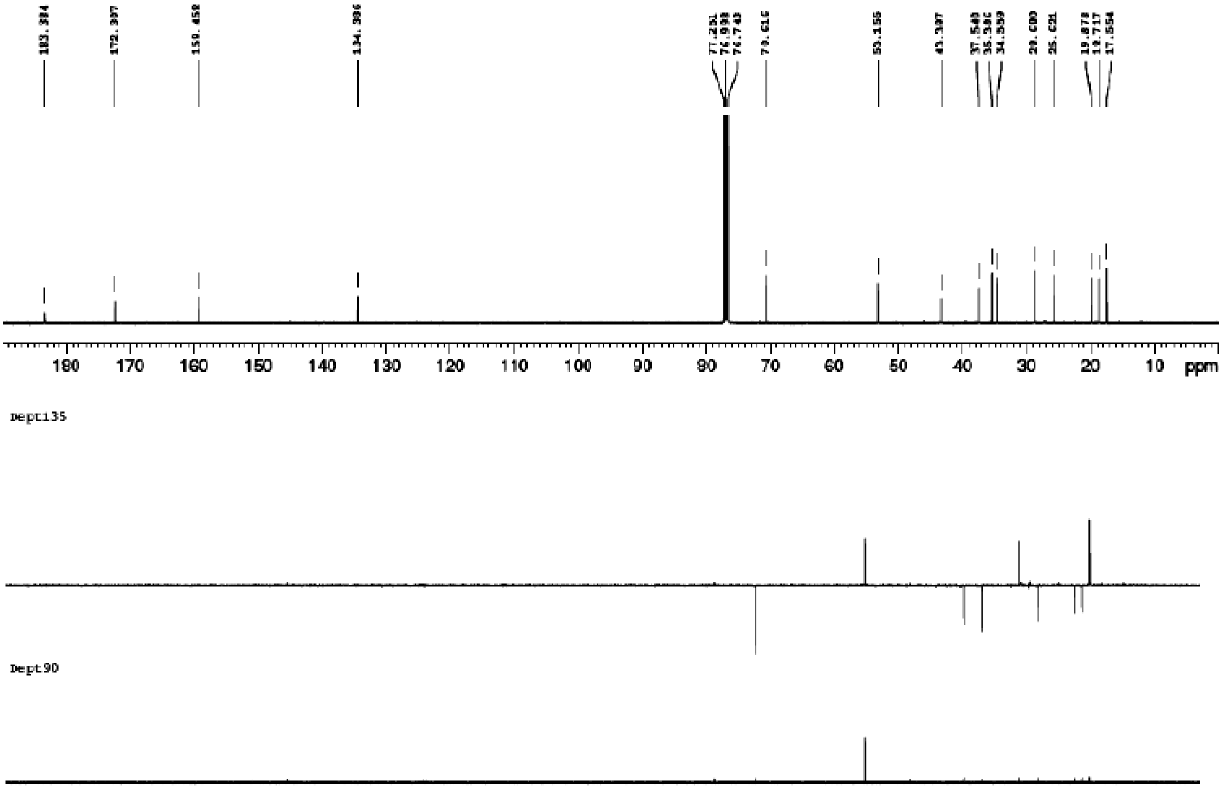

Figure S9. ^{13}C spectrum of **2**

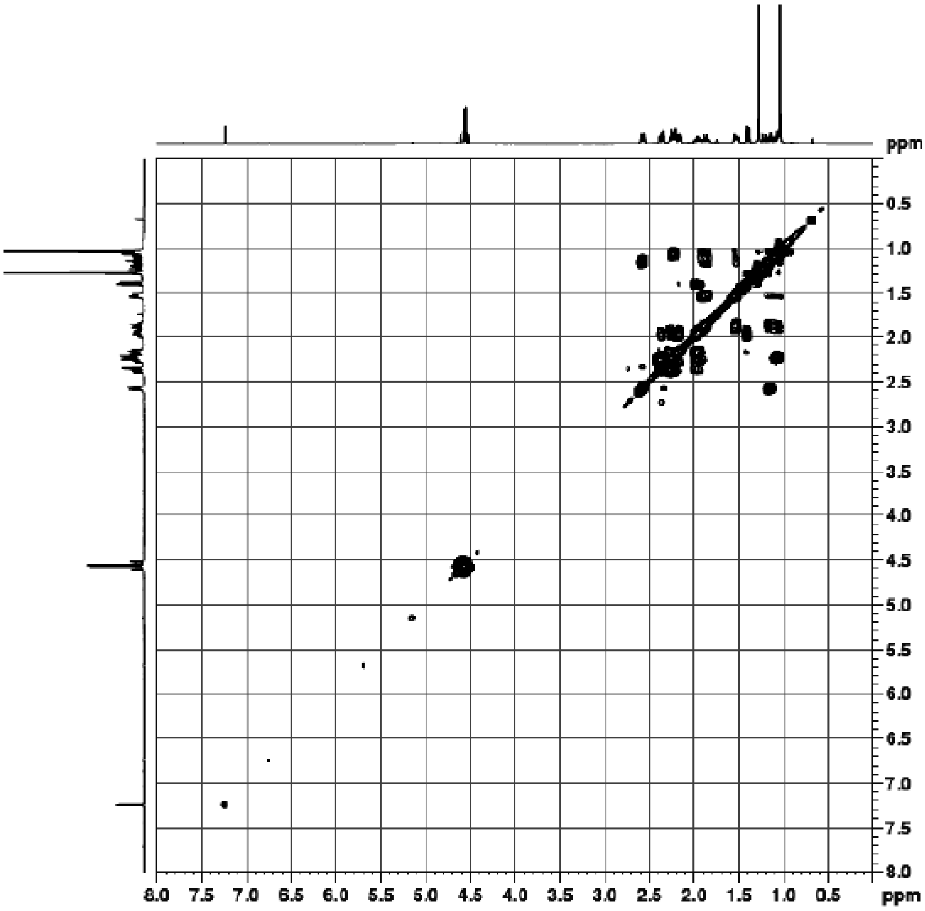

Figure S10. COSY spectrum of **2**

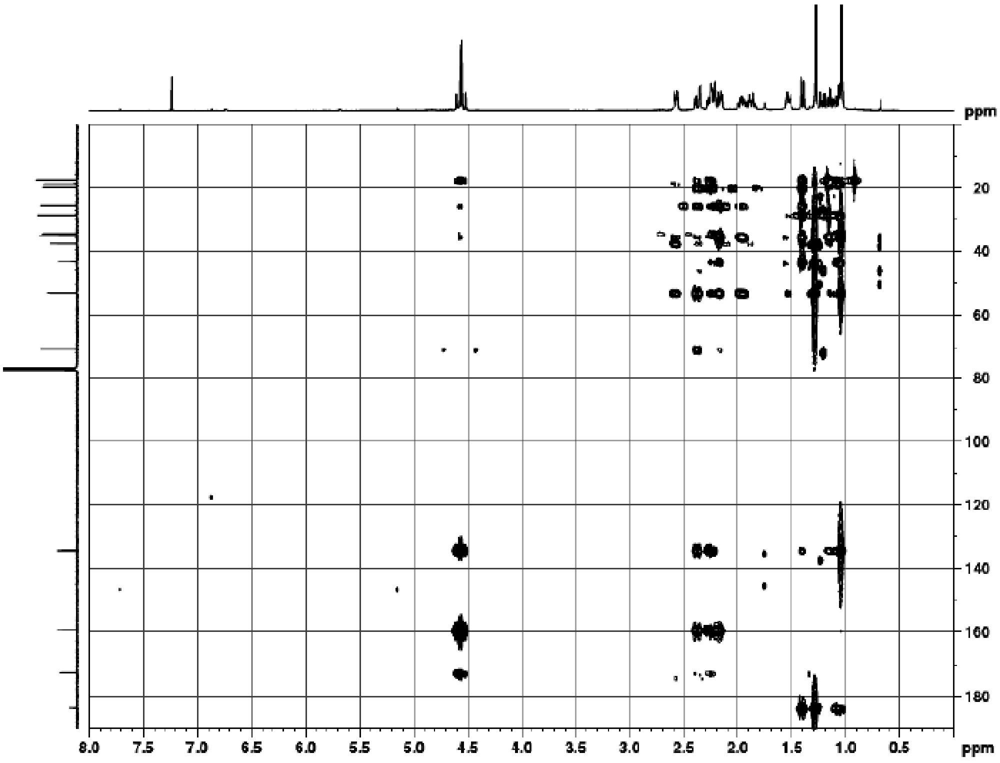

Figure S11. HMBC spectrum of **2**

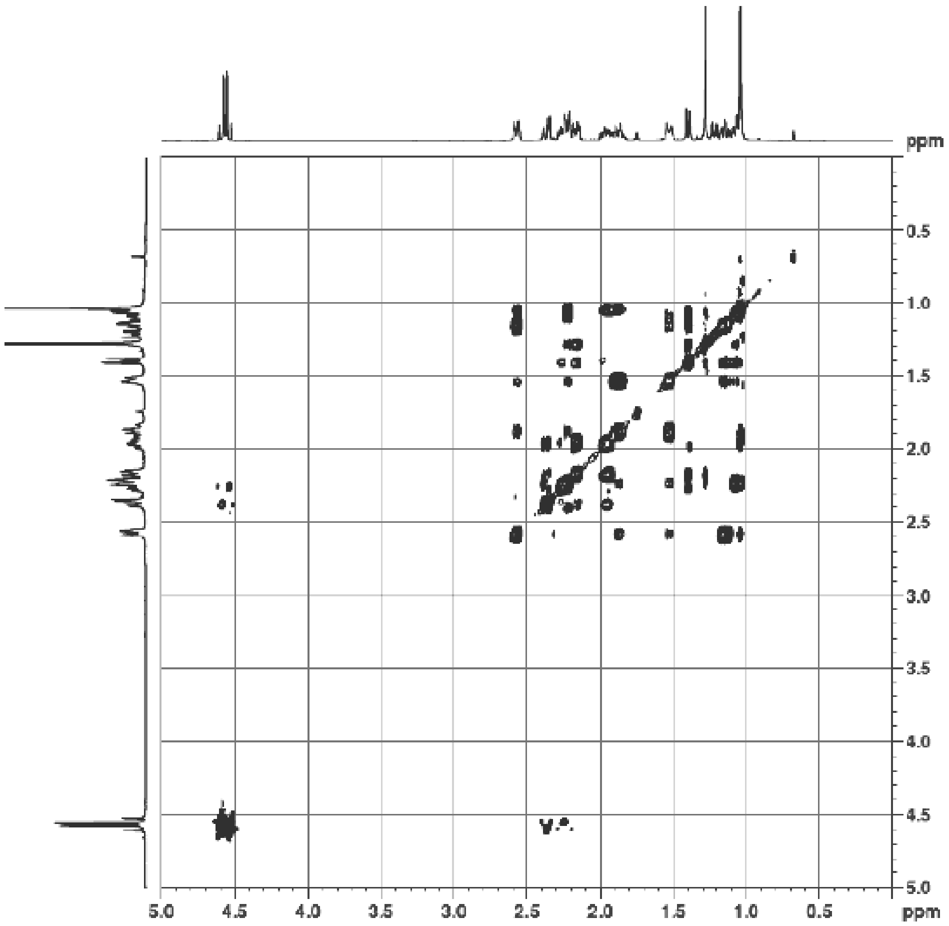

Figure S12. NOESY spectrum of **2**

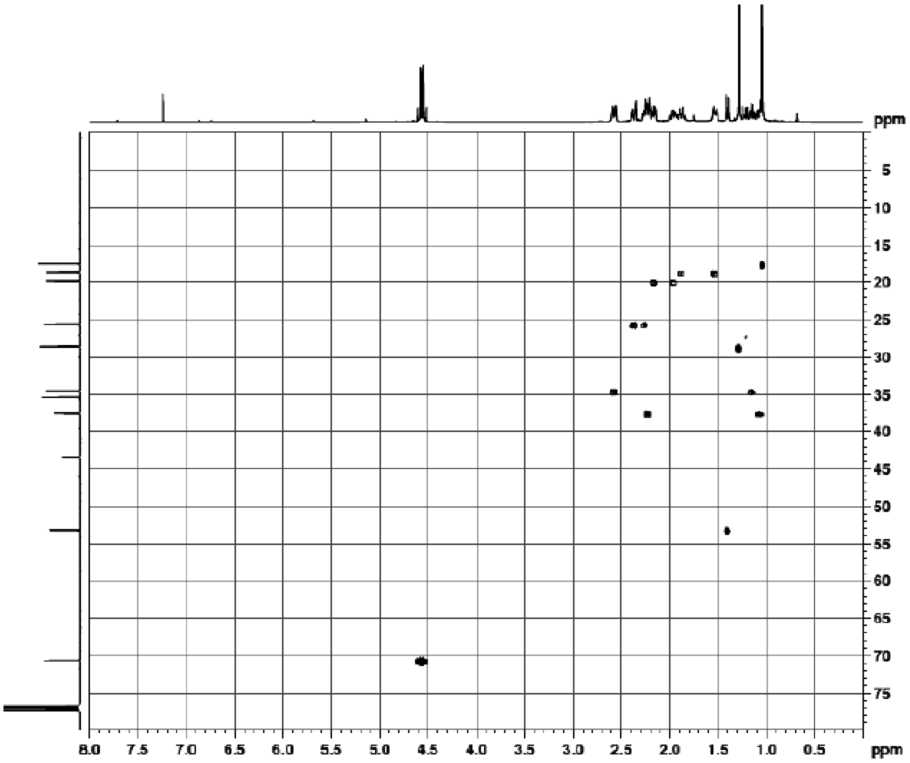

Figure S13. HSQC spectrum of **2**

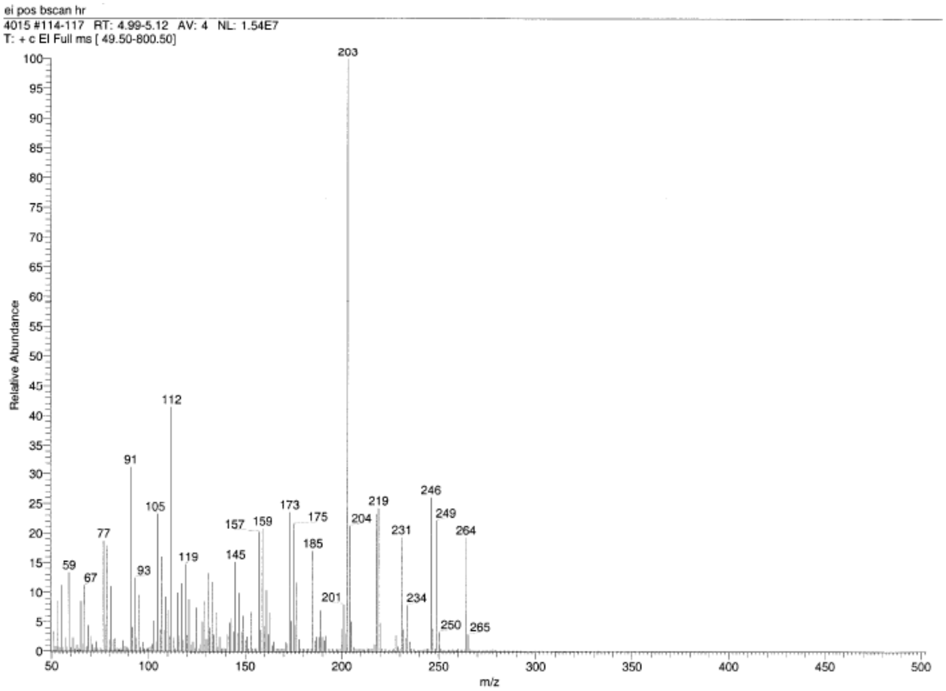

Figure S14. EI-MS spectrum of **2**

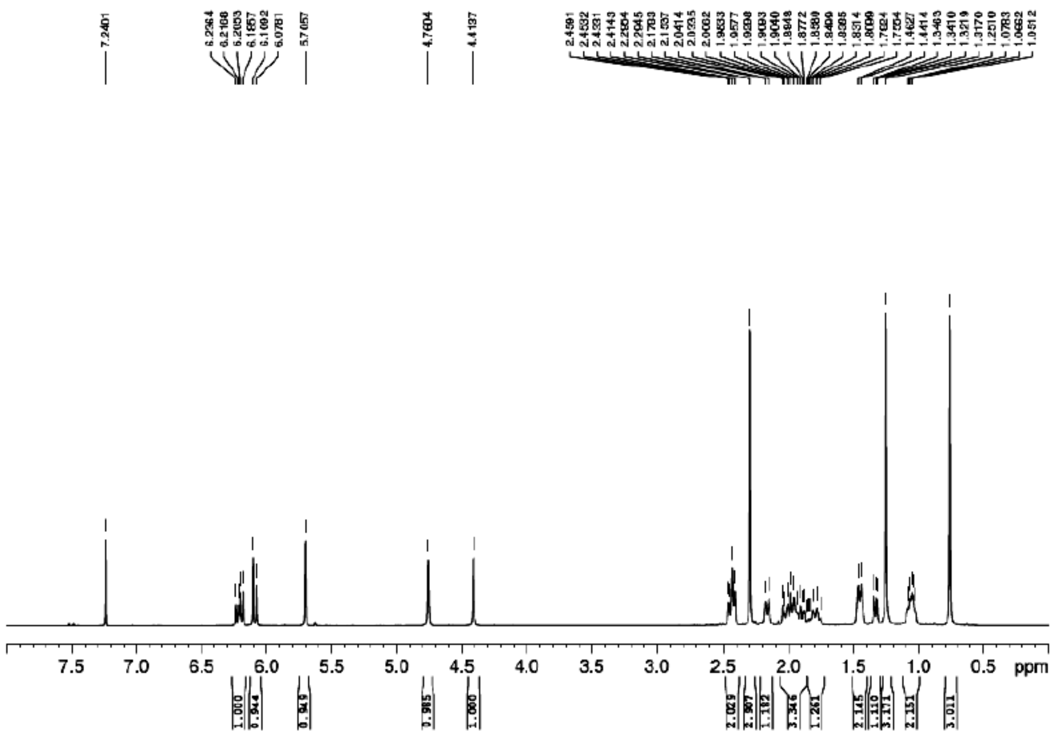

Figure S15. ^{1}H NMR spectrum of **3**

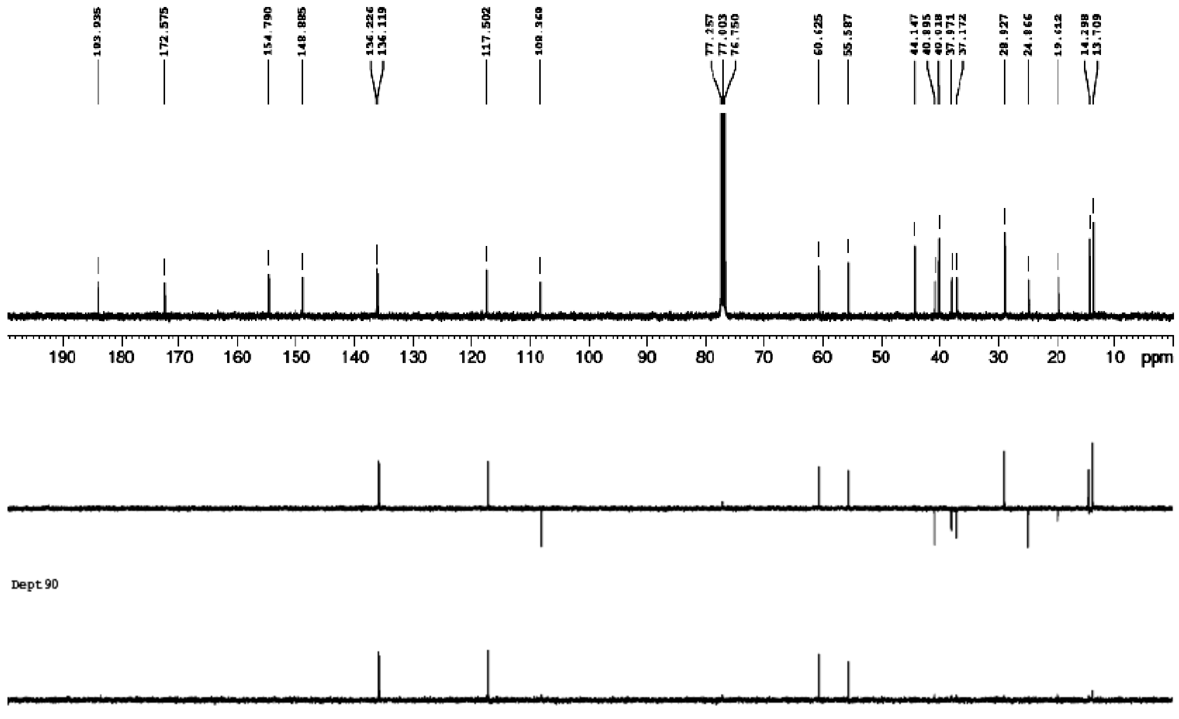

Figure S16. ^{13}C NMR spectrum of **3**

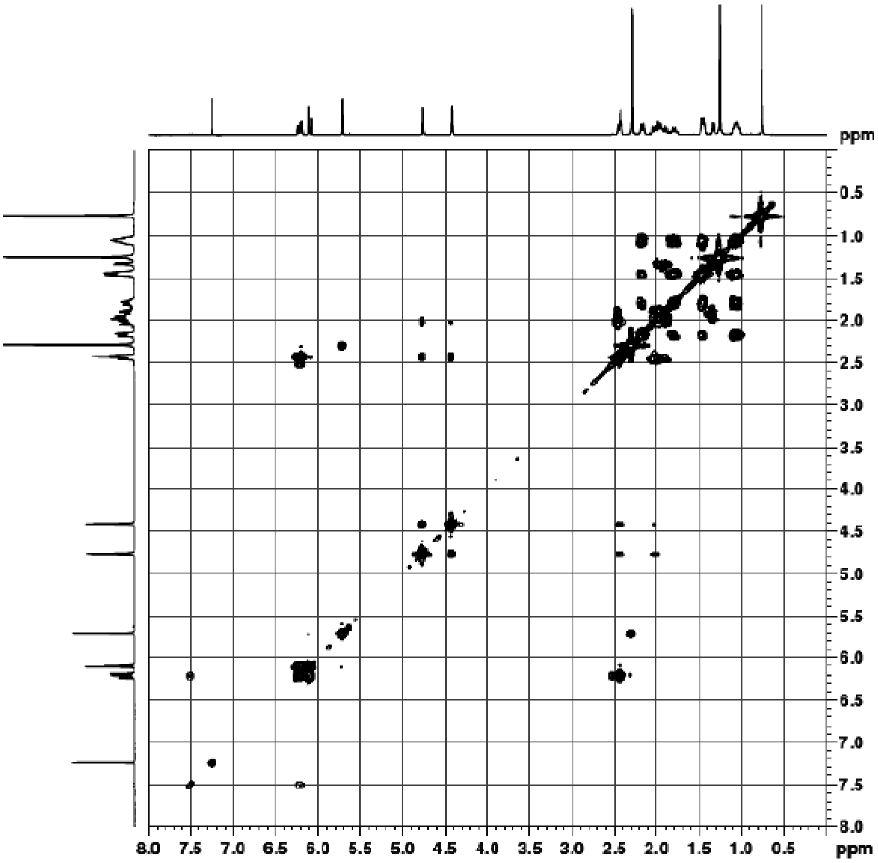

Figure S17. COSY spectrum of **3**

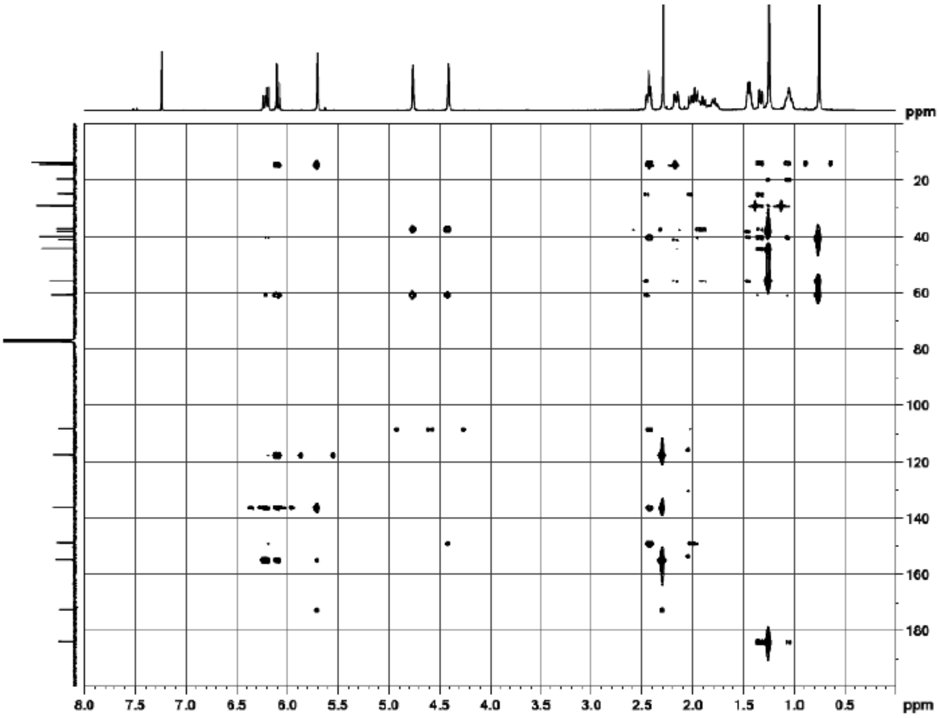

Figure S18. HMBC spectrum of **3**

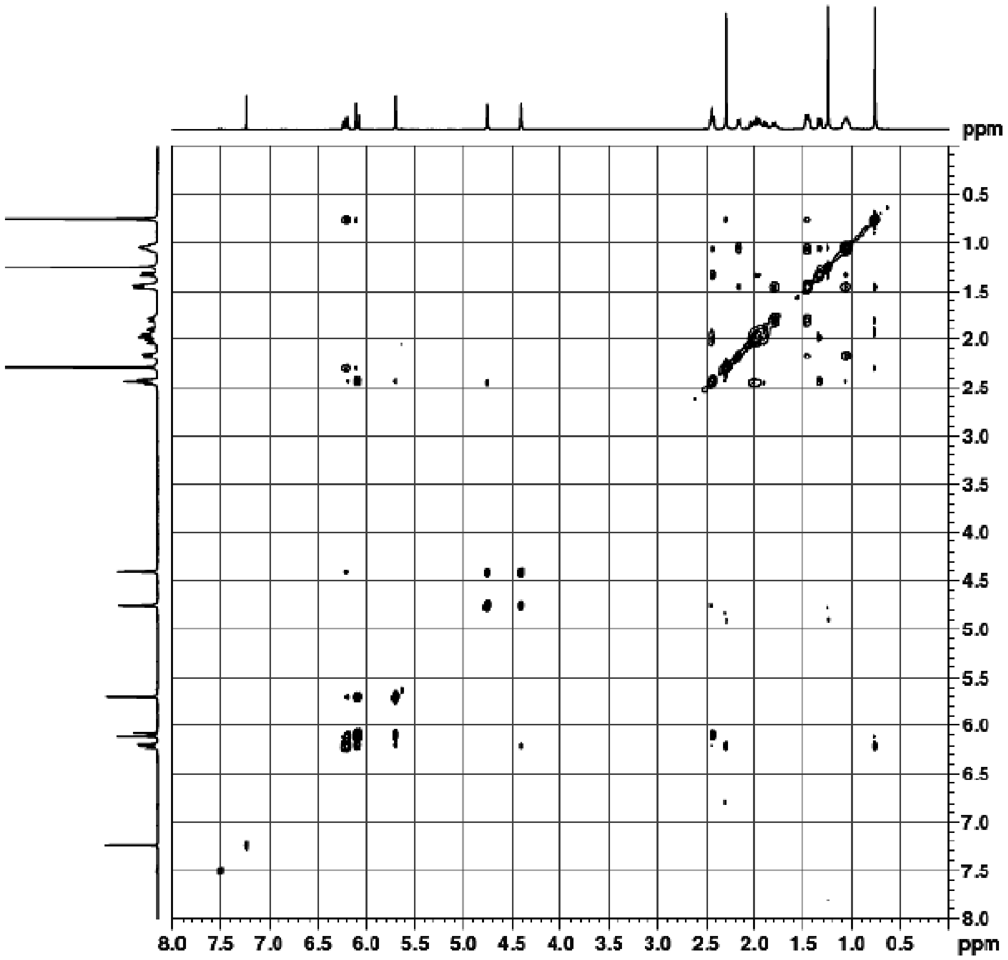

Figure S19. NOESY spectrum of **3**

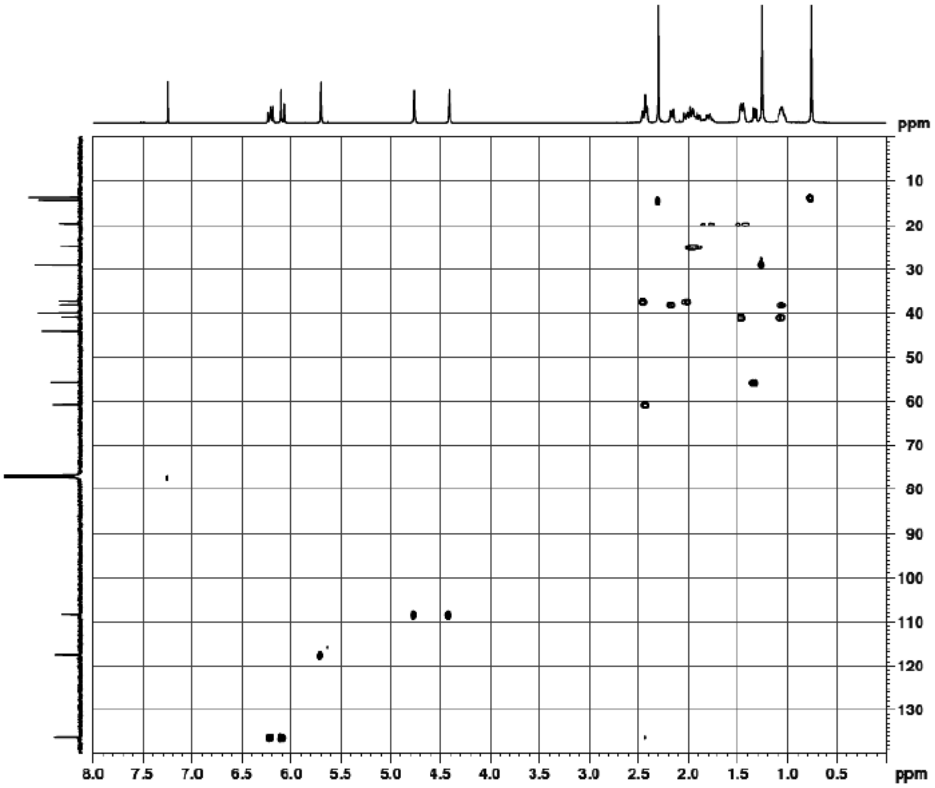

Figure S20. HSQC spectrum of **3**

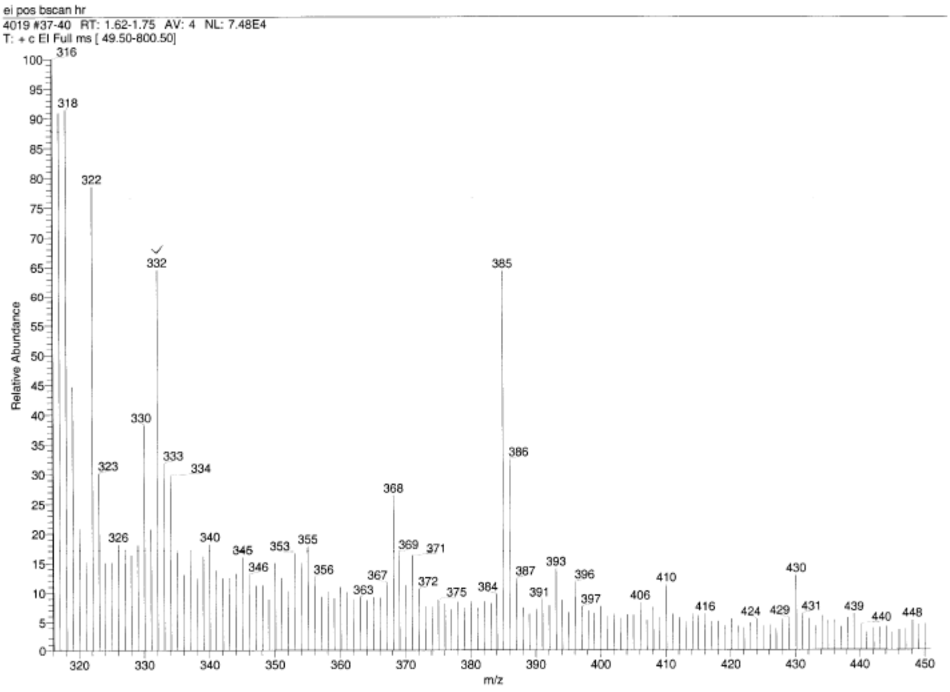

Figure S21. EI-MS spectrum of **3**

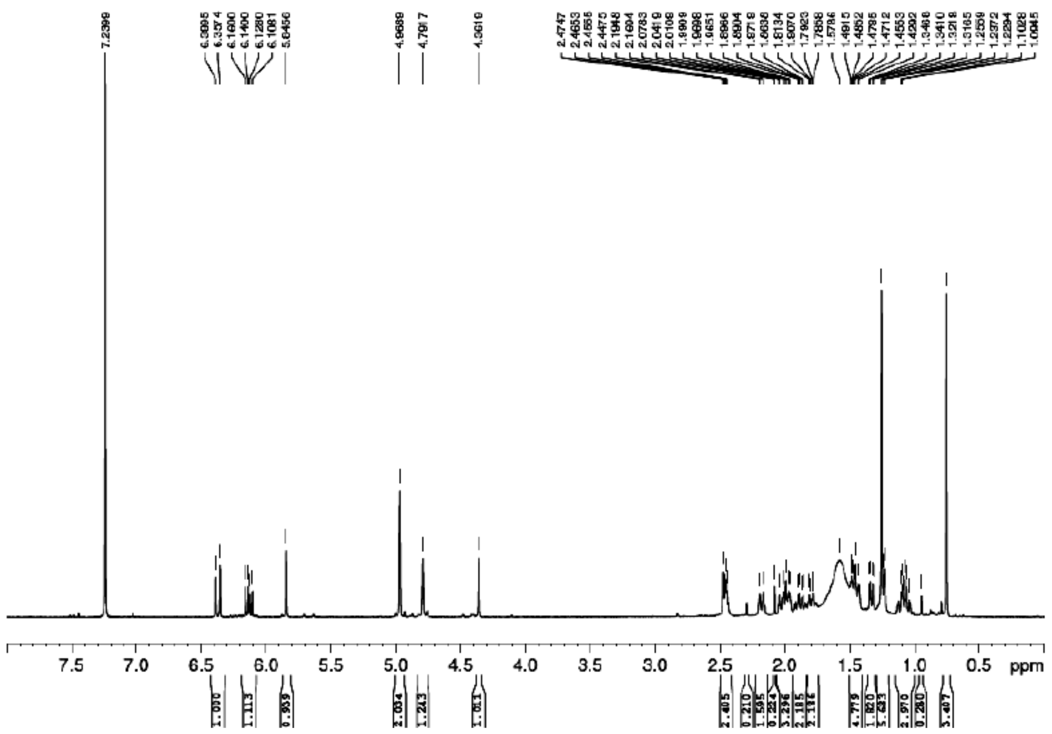

Figure S22. ^{1}H NMR spectrum of **4**

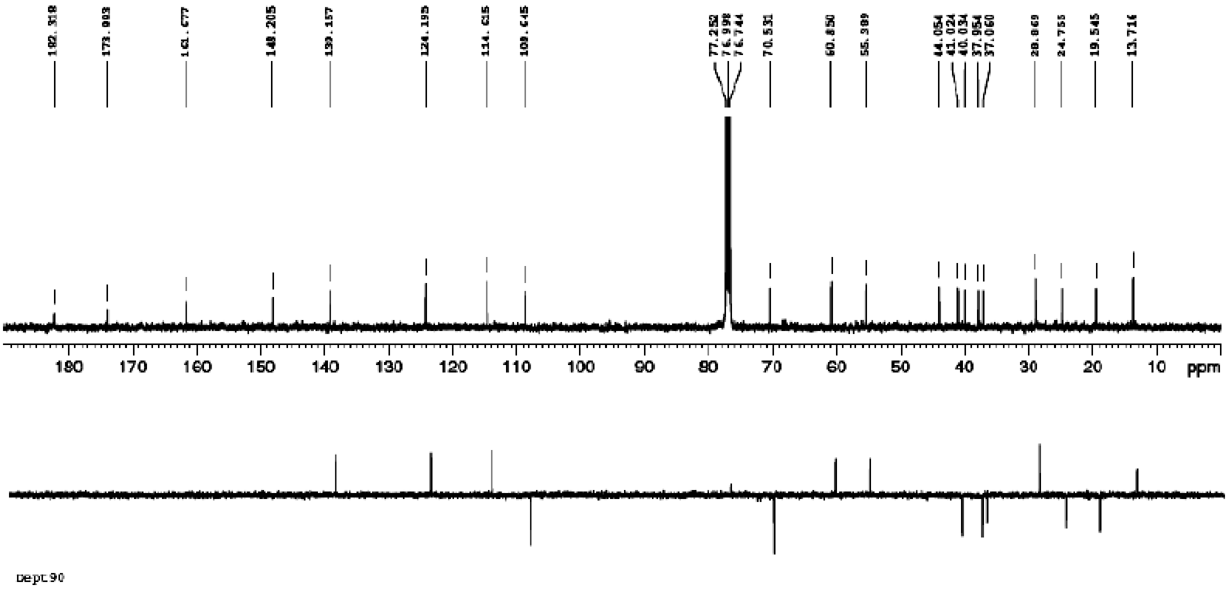

Figure S23. ^{13}C spectrum of **4**

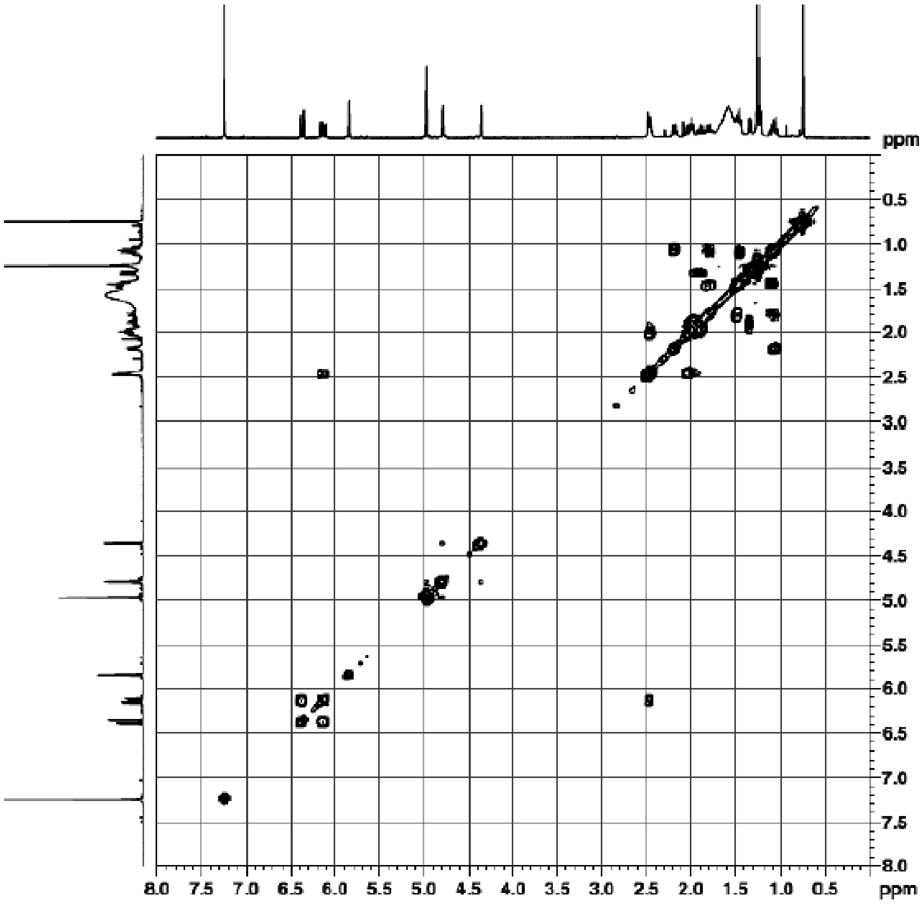

Figure S24. COSY spectrum of **4**

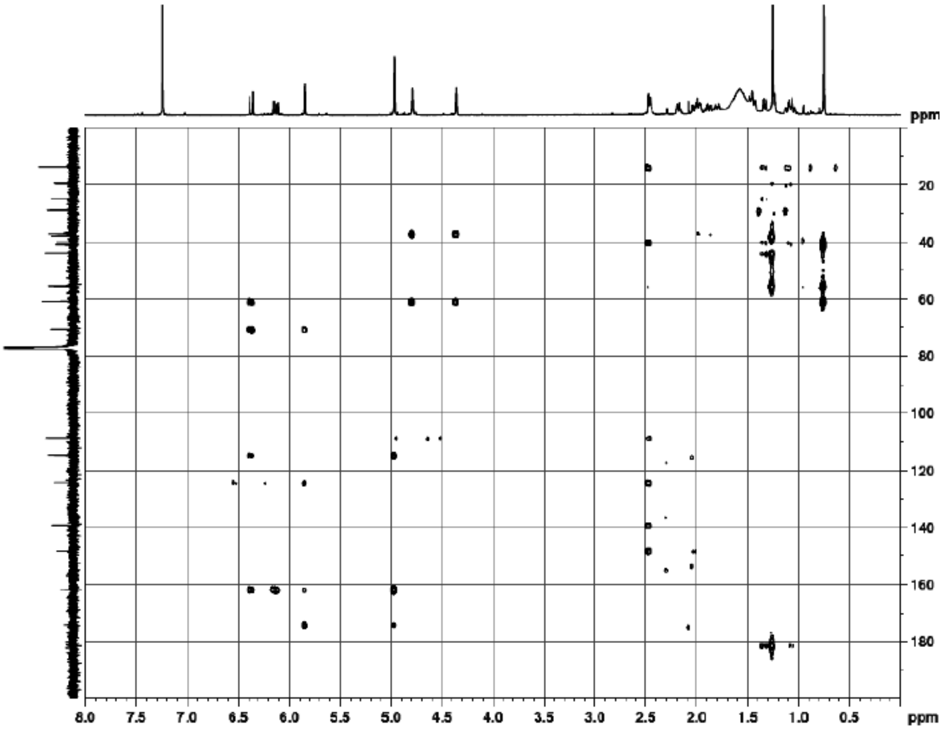

Figure S25. HMBC spectrum of **4**

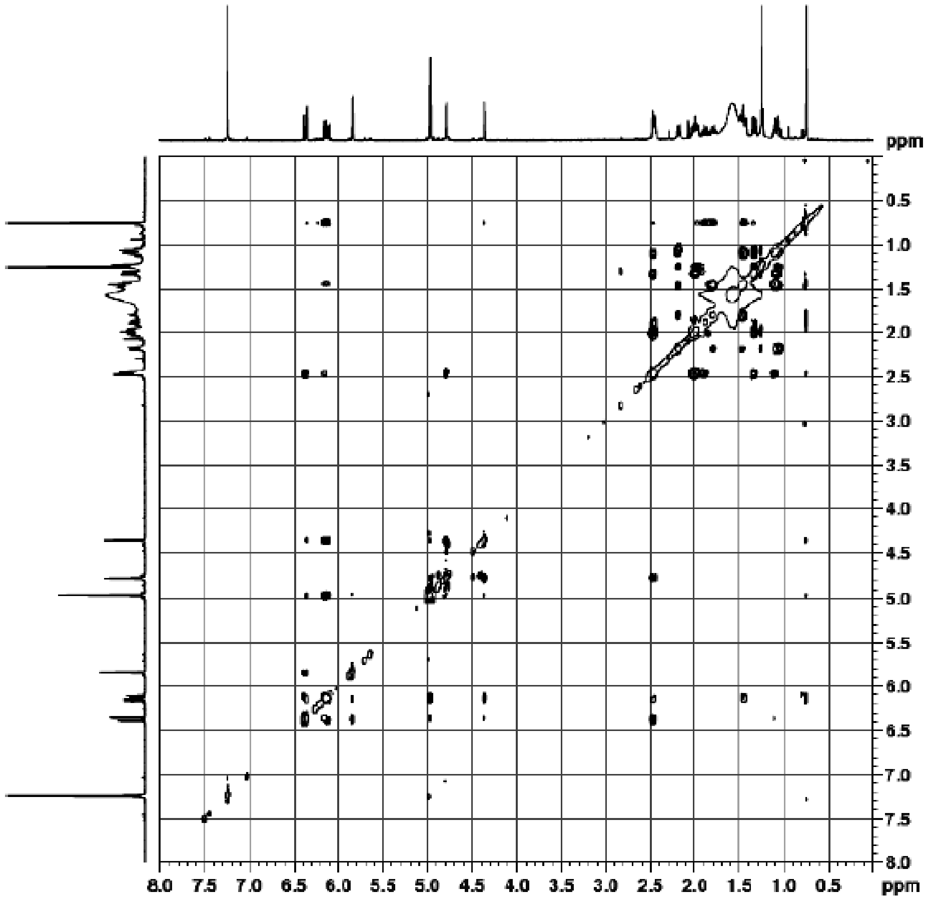

Figure S26. NOESY spectrum of **4**

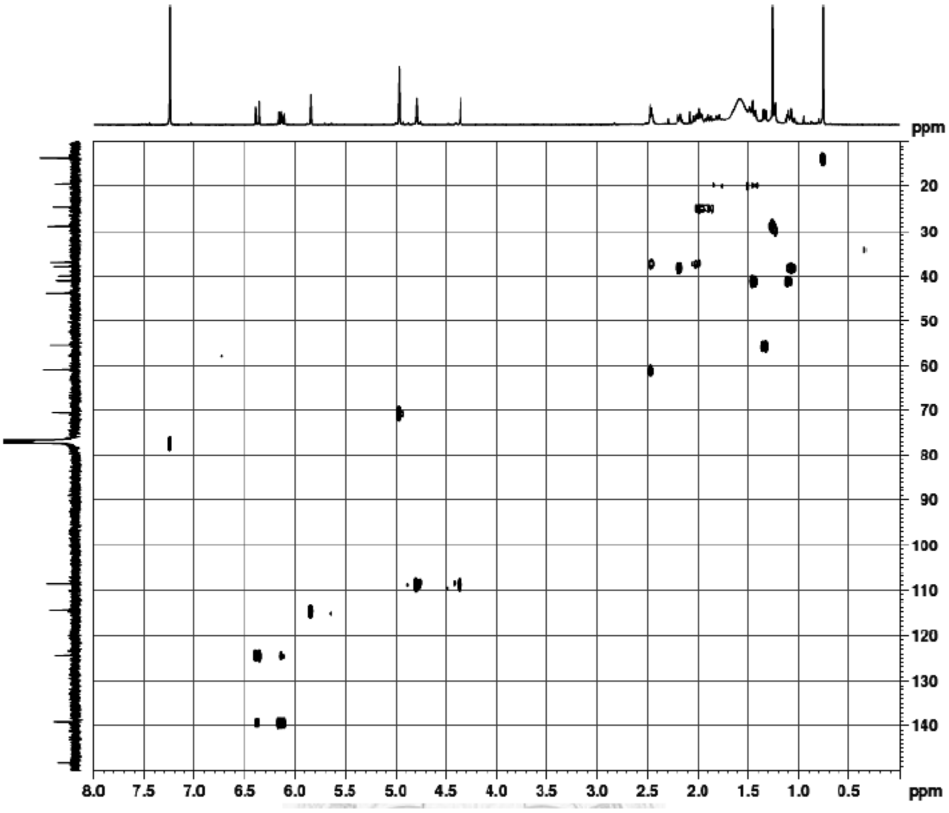

Figure S27. HSQC spectrum of **4**

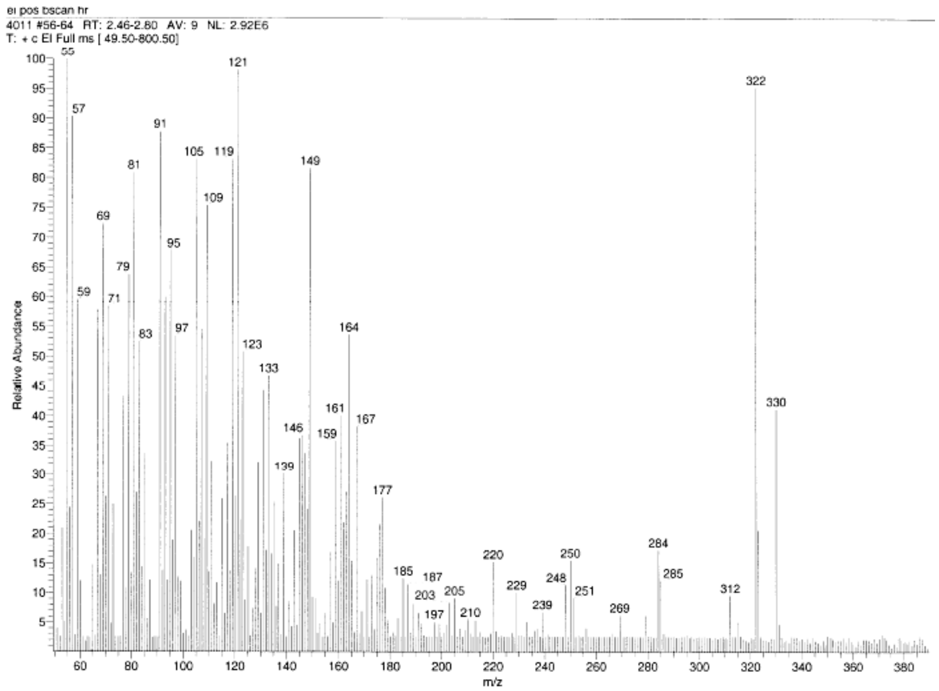

Figure S28. EI-MS spectrum of **4**

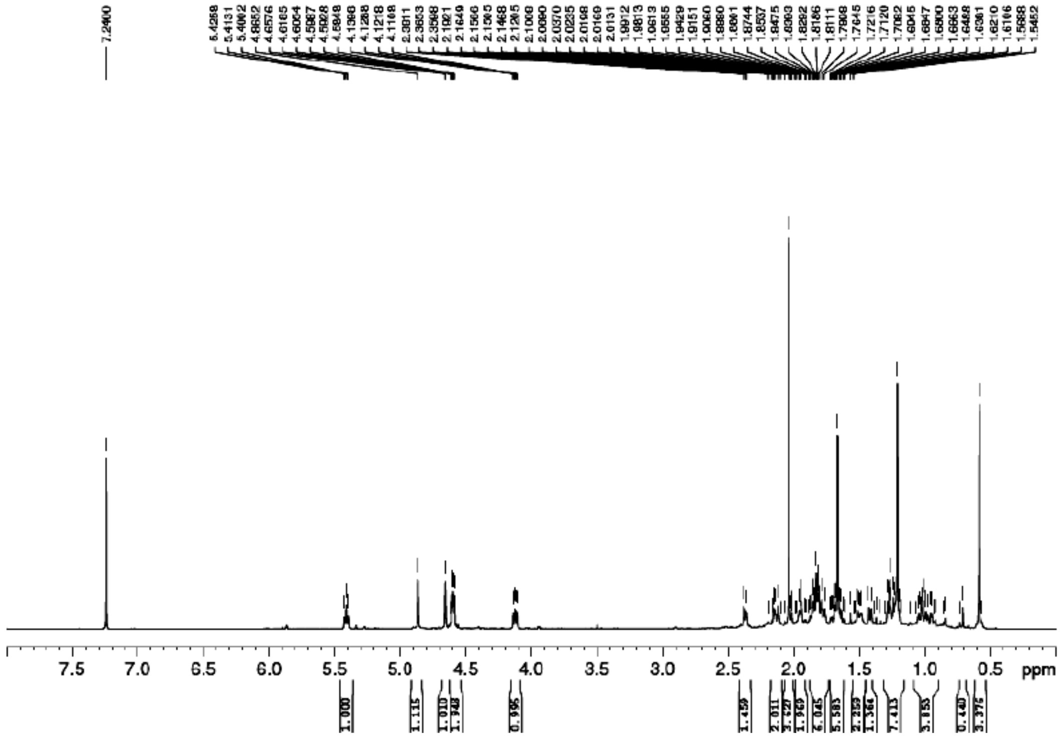

Figure S29. ^{1}H NMR spectrum of **5**

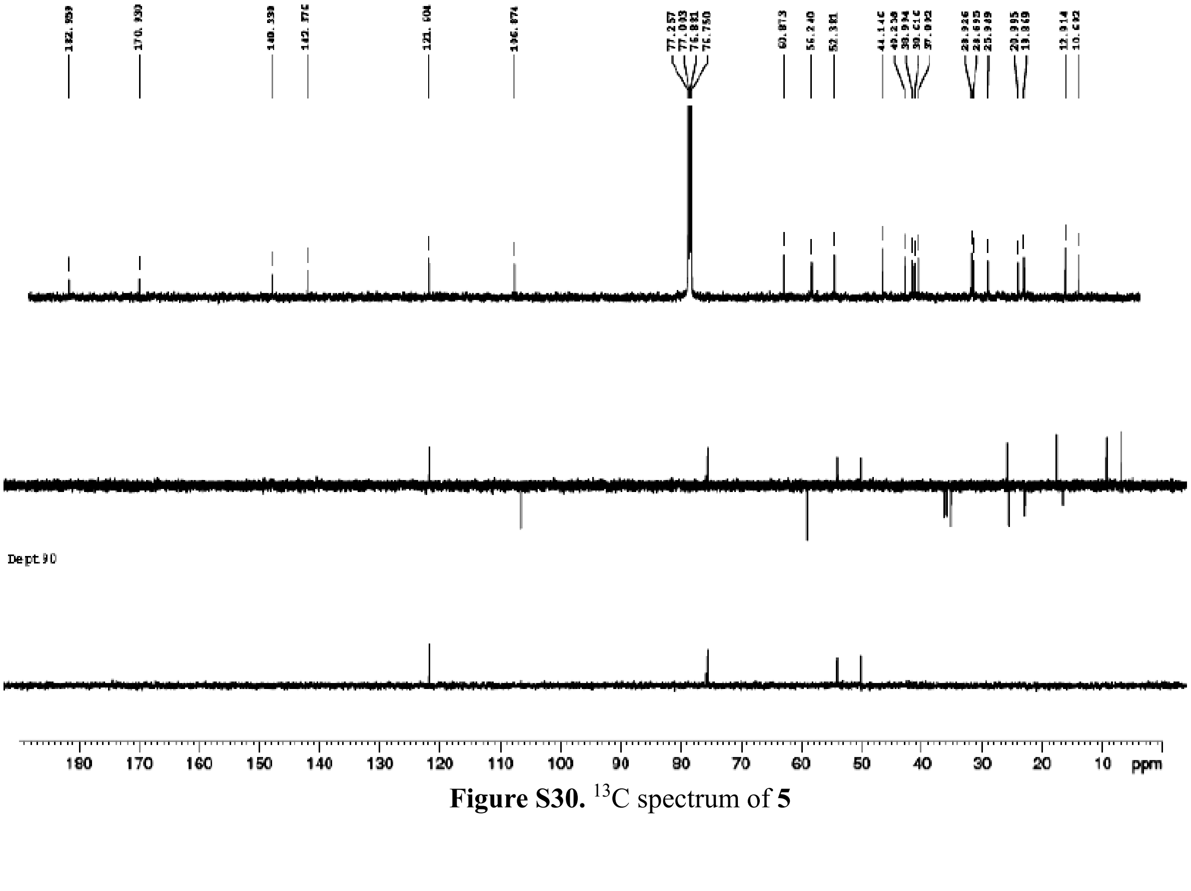

Figure S30. ^{13}C spectrum of **5**

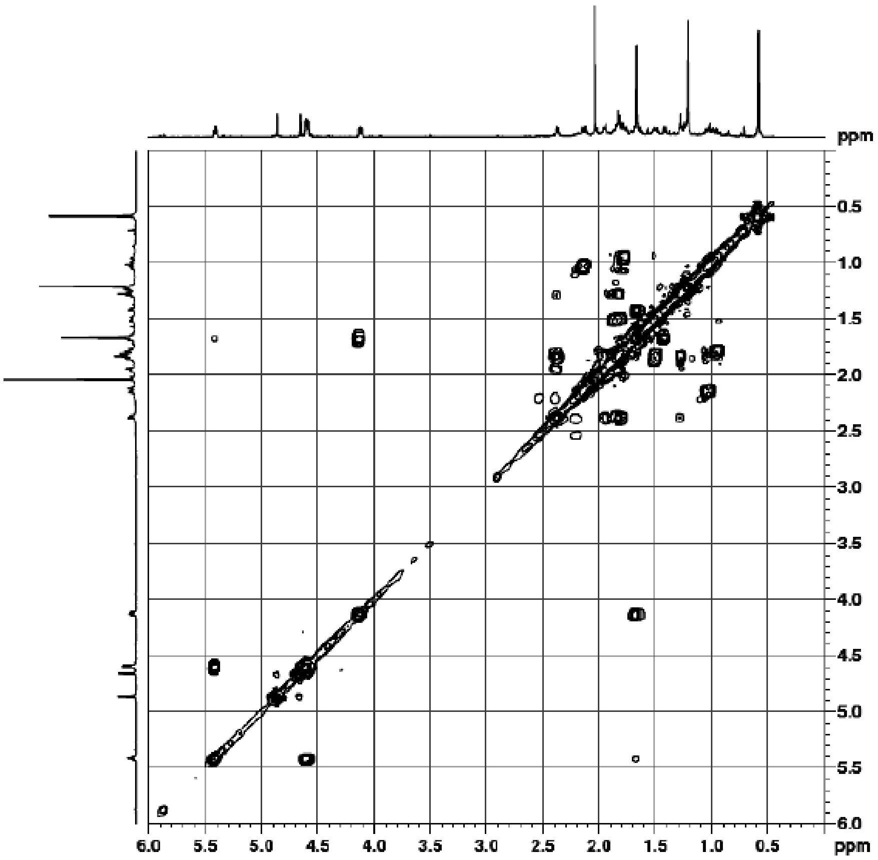

Figure S31. COSY spectrum of **5**

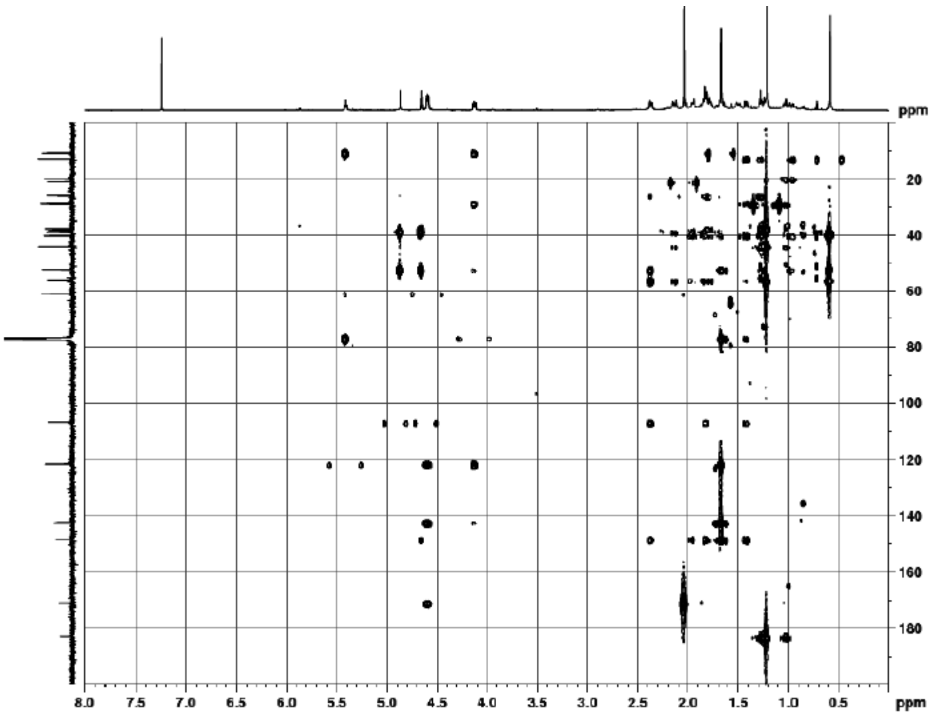

Figure S32. HMBC spectrum of **5**

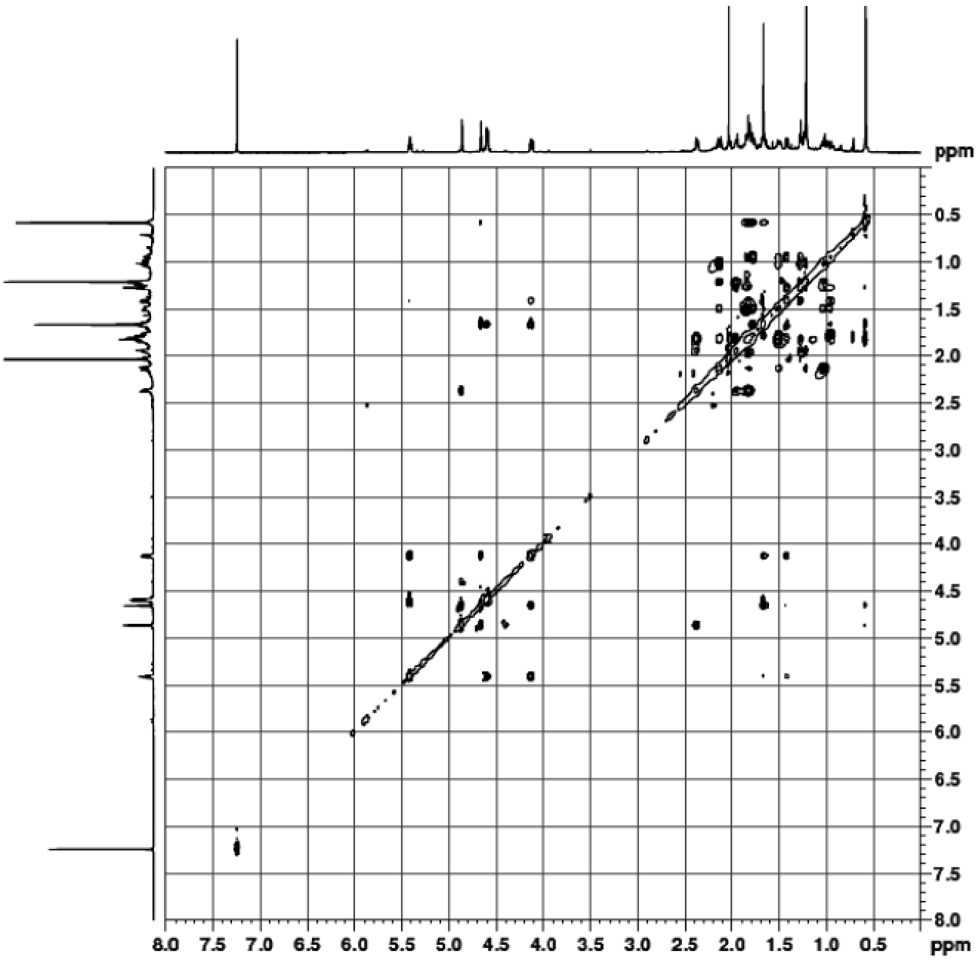

Figure S33. NOESY spectrum of **5**

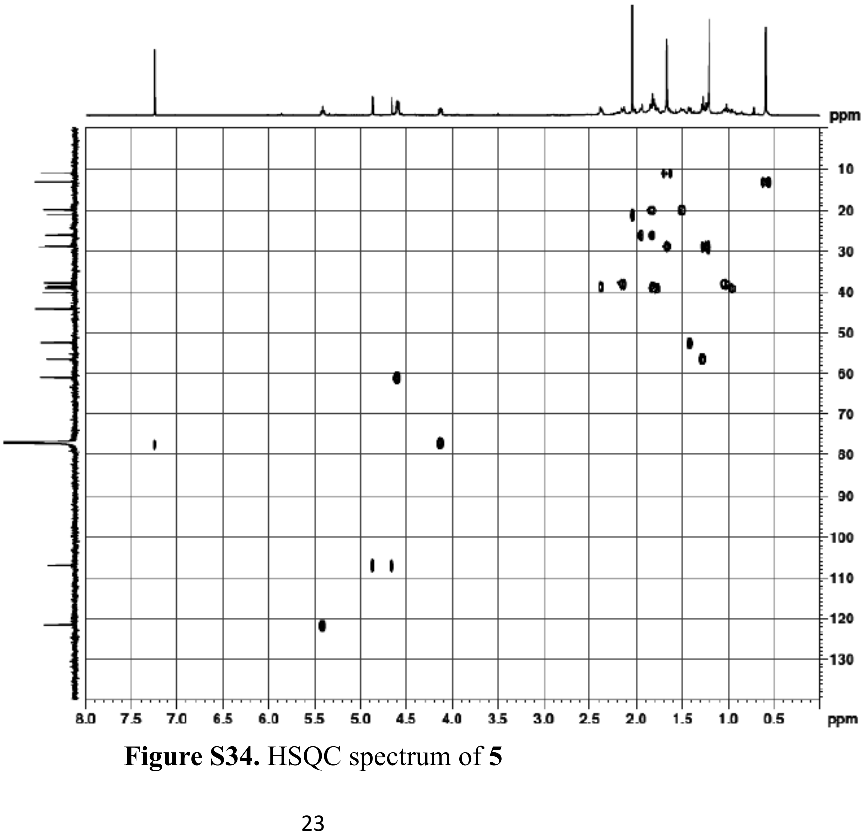

Figure S34. HSQC spectrum of **5**

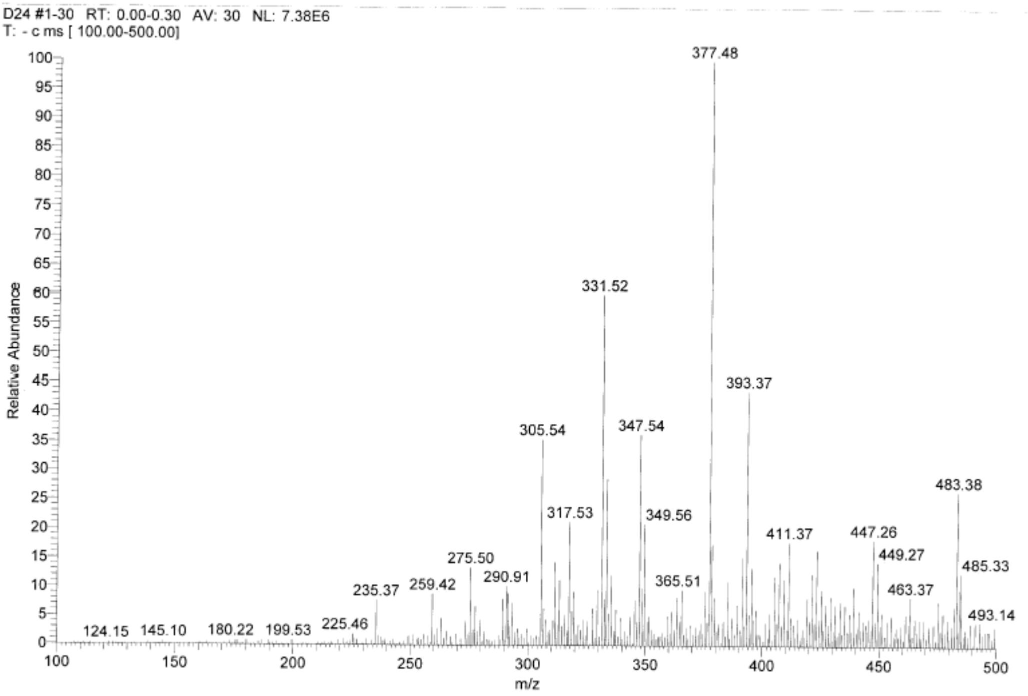

Figure S35. EI-MS spectrum of **5**

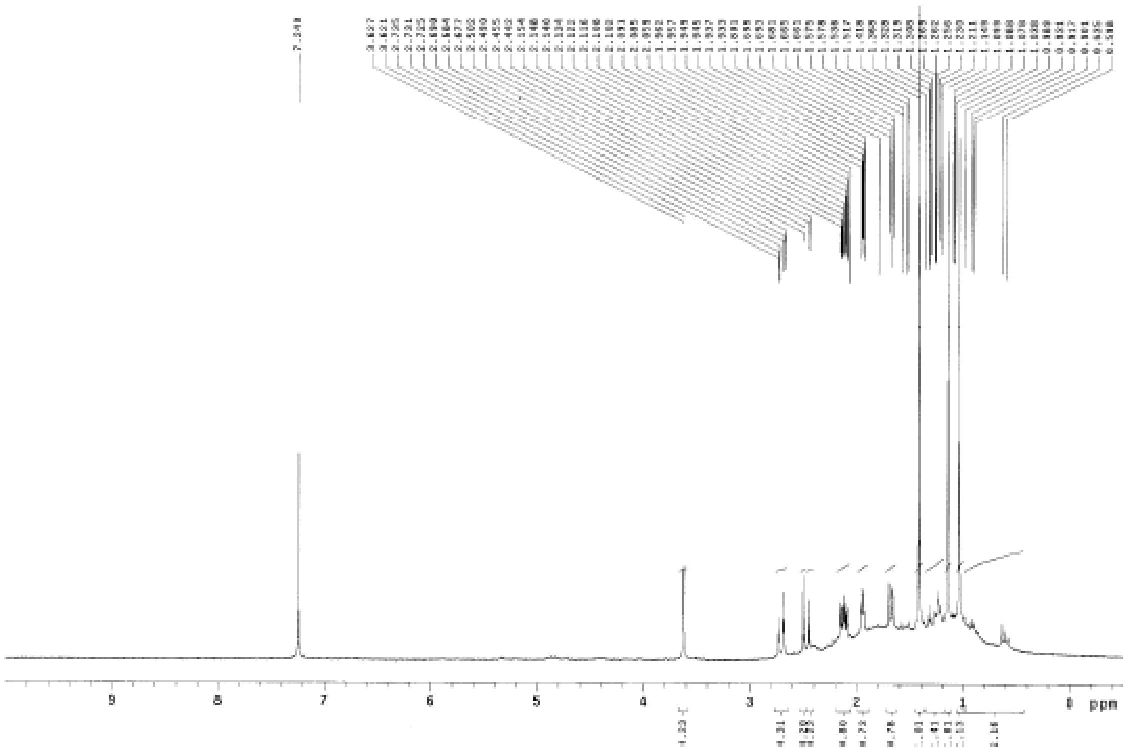

Figure S36. ^{1}H NMR spectrum of **6**

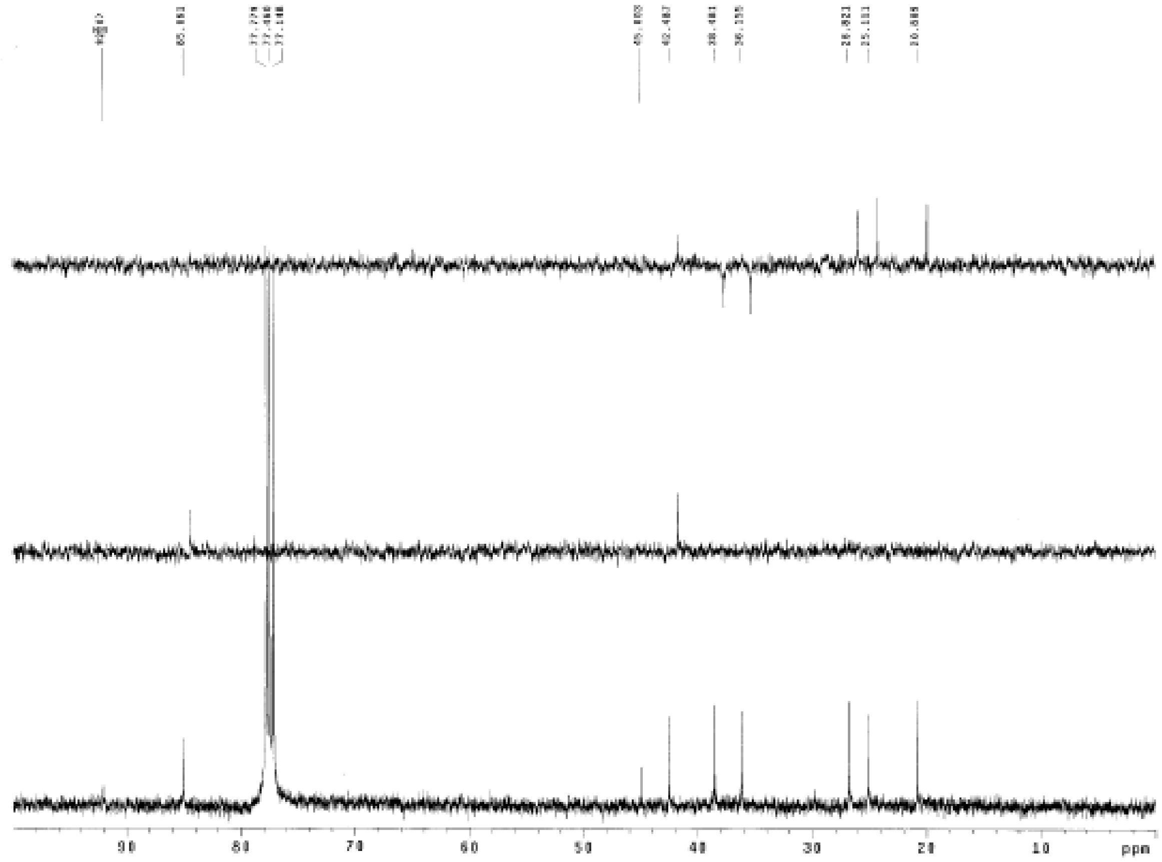

Figure S37. ^{13}C NMR/DEPT spectra of **6**

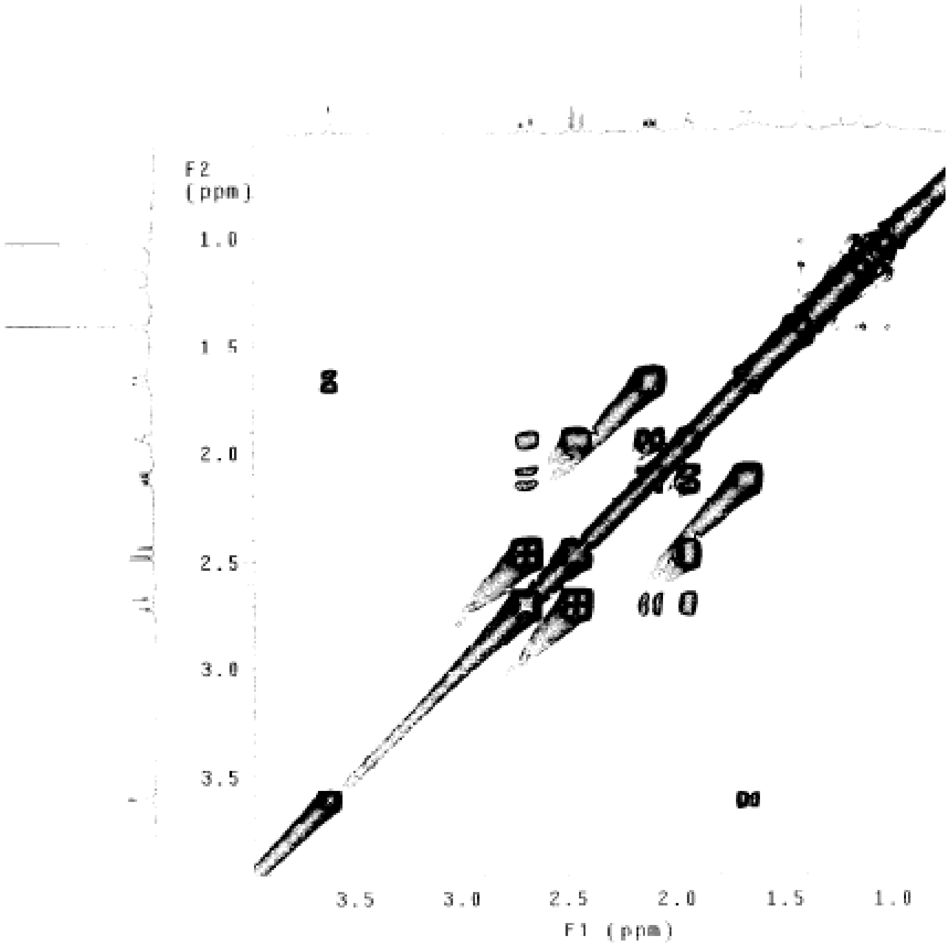

Figure S38. COSY spectrum of **6**

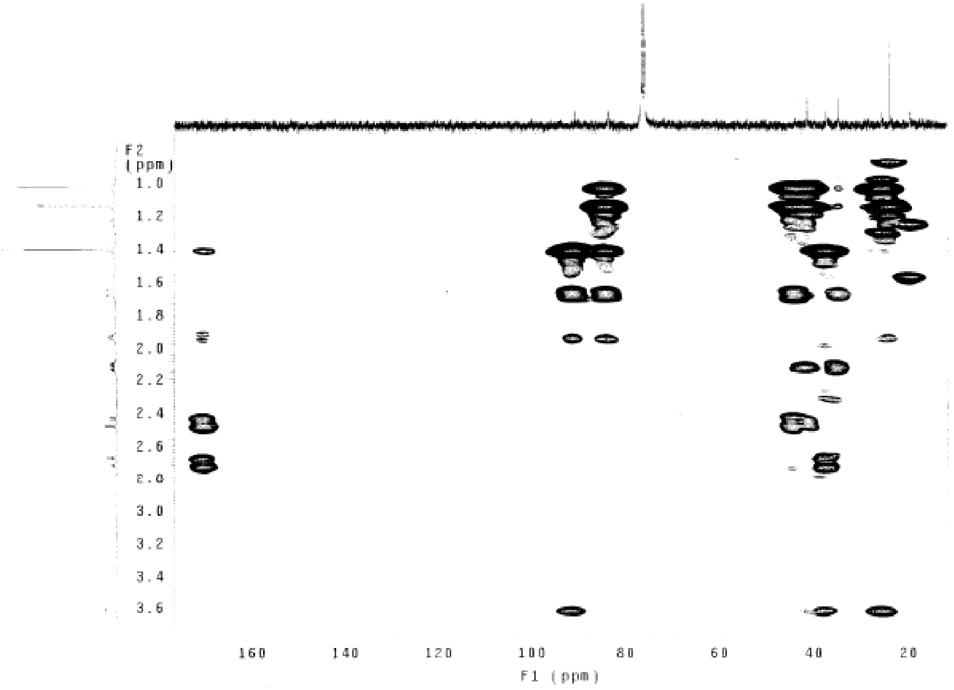

Figure S39. HMBC spectrum of **6**

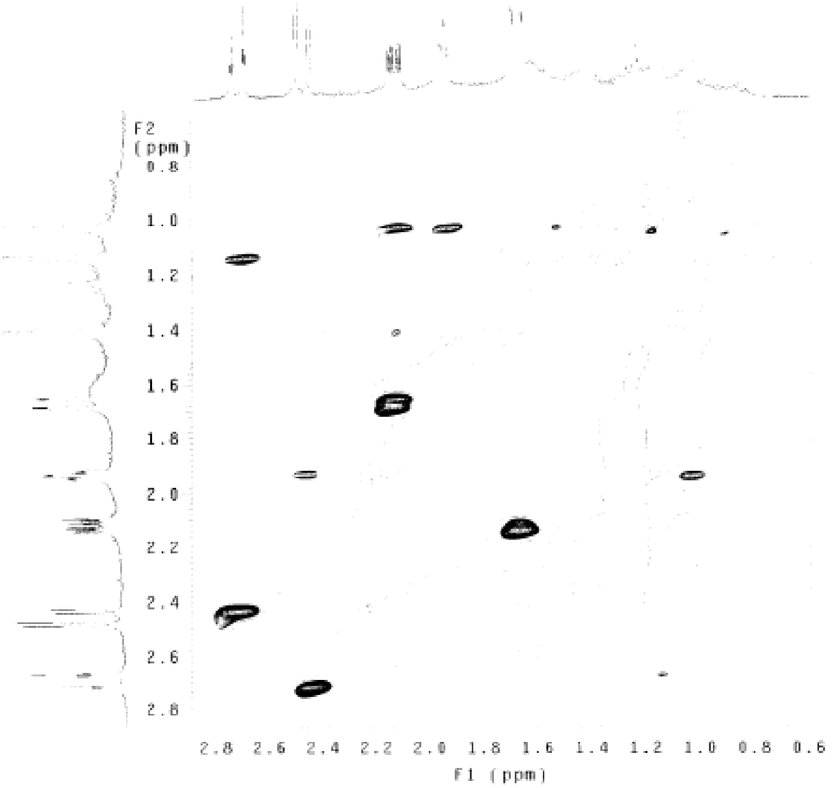

Figure S40. NOESY spectrum of **6**

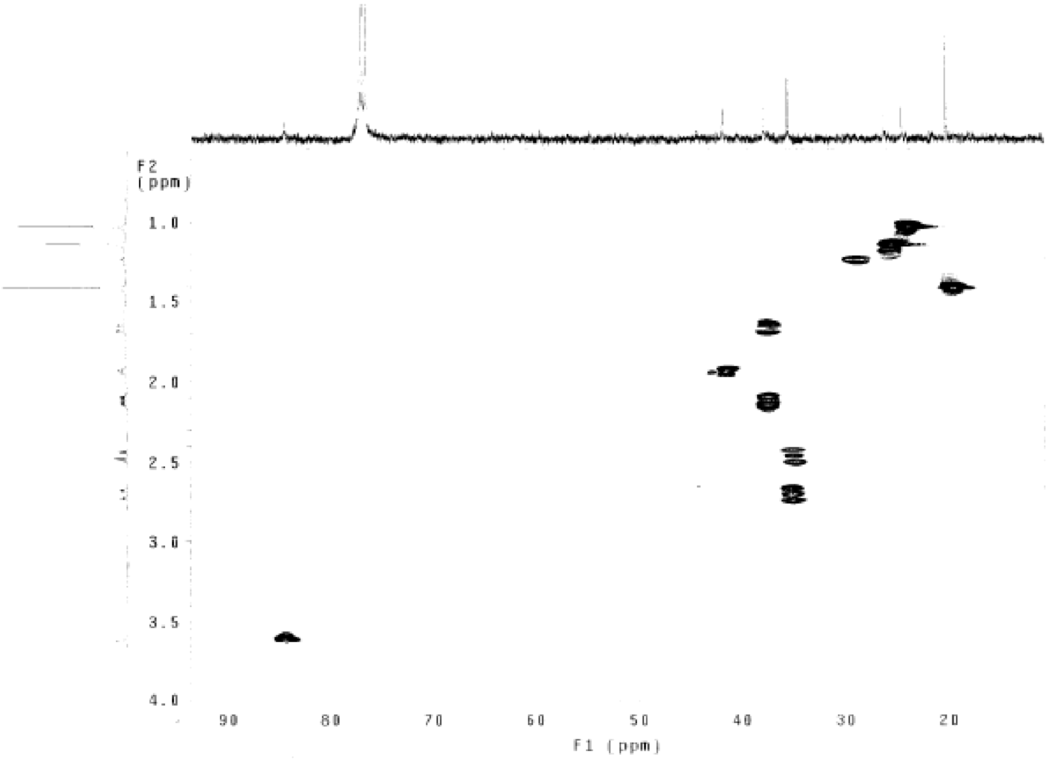

Figure S41. HSQC spectrum of **6**

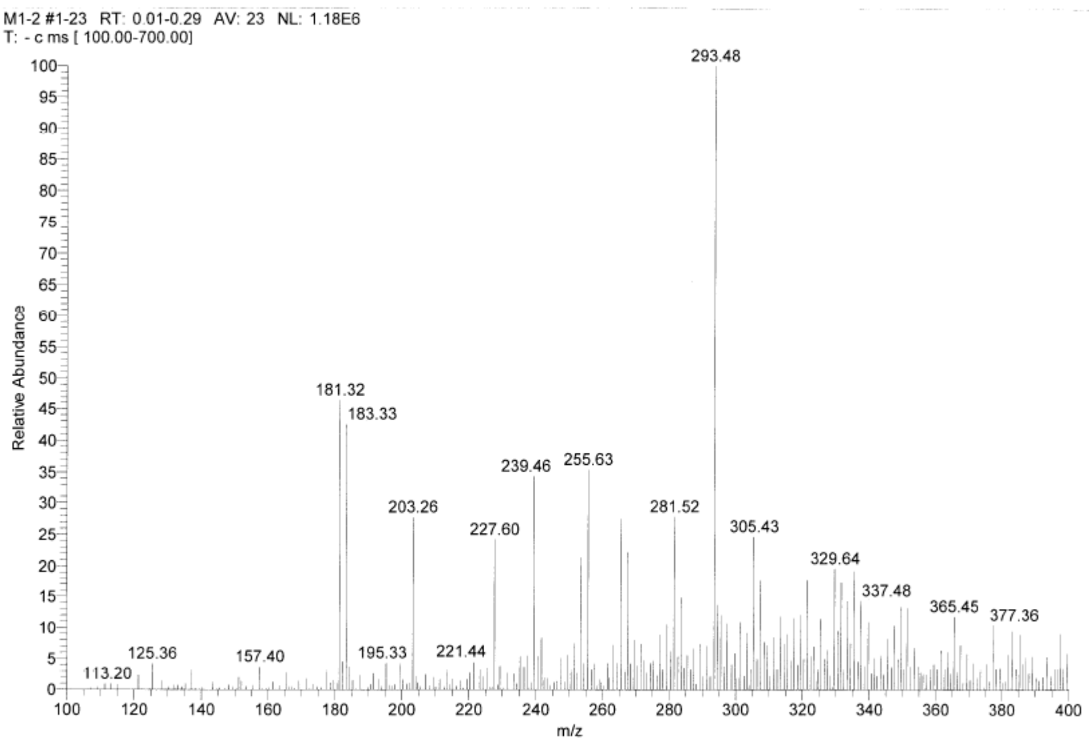

Figure S42. EI-MS spectrum of **6**

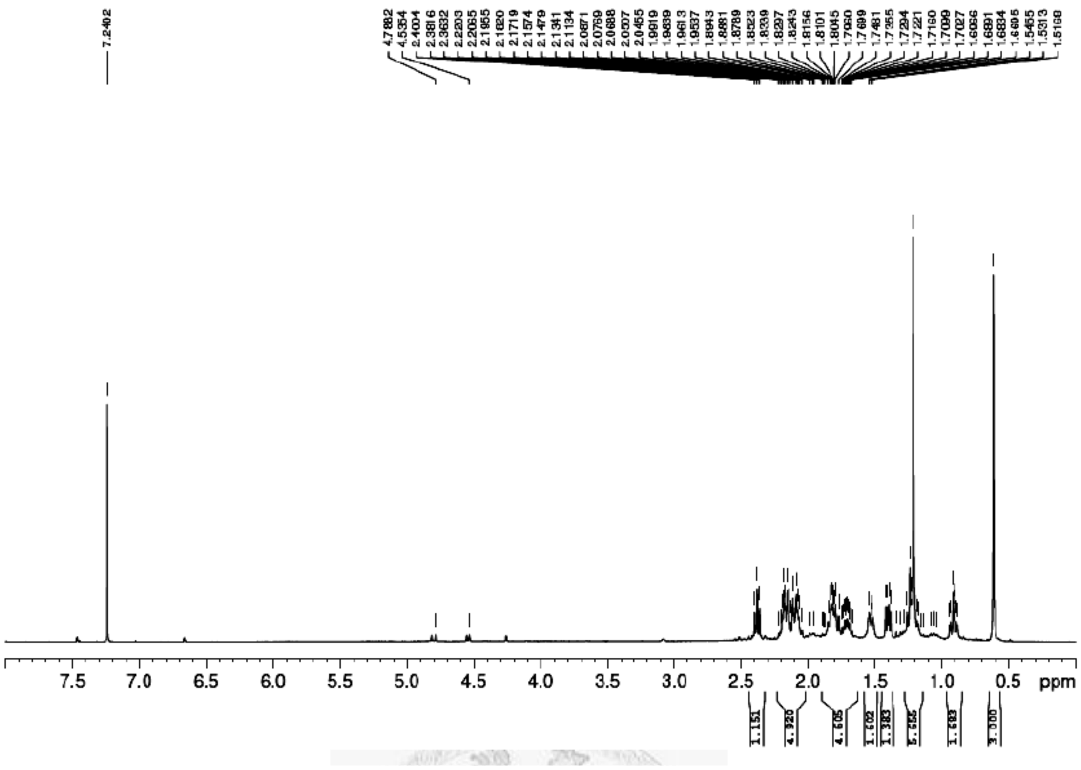

Figure S43. ^{1}H NMR spectrum of **7**

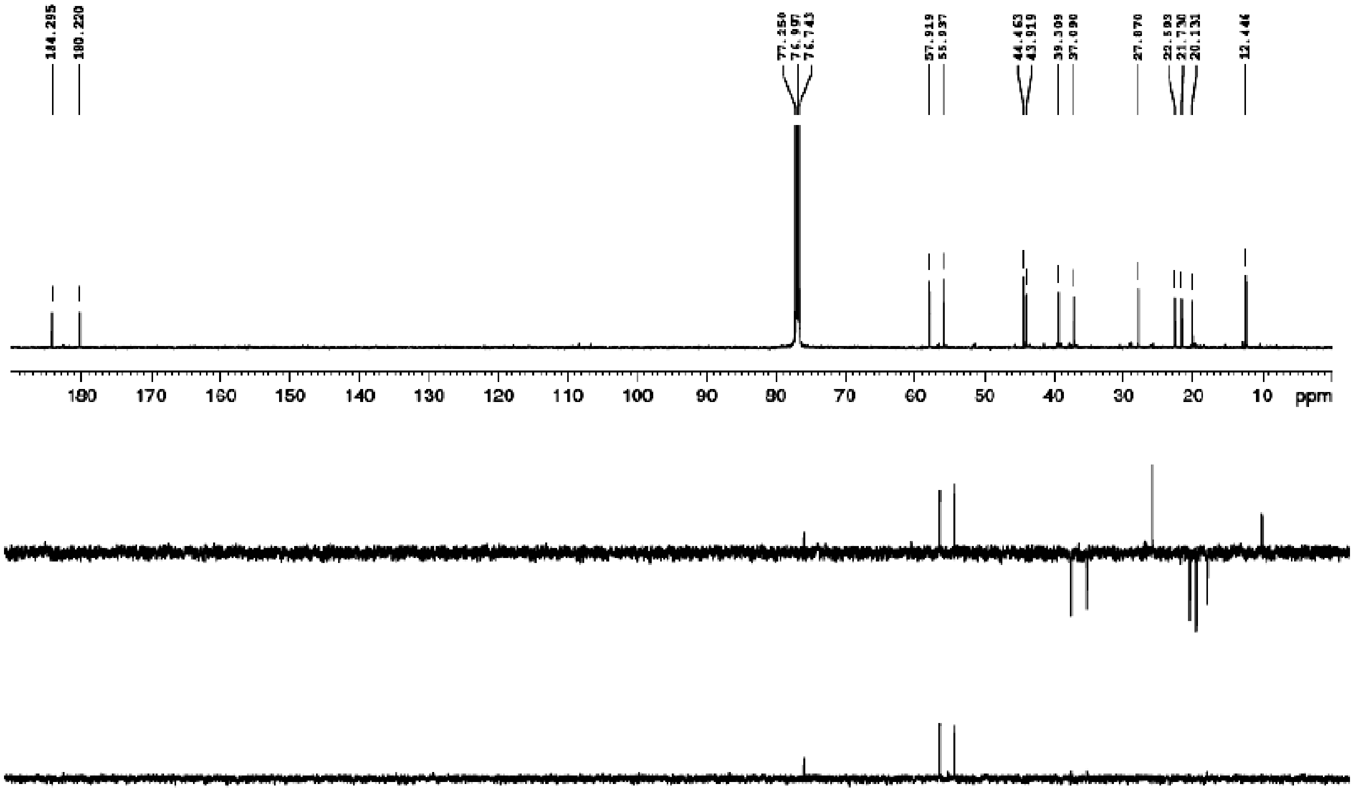

Figure S44. ^{13}C NMR/DEPT spectra of **7**

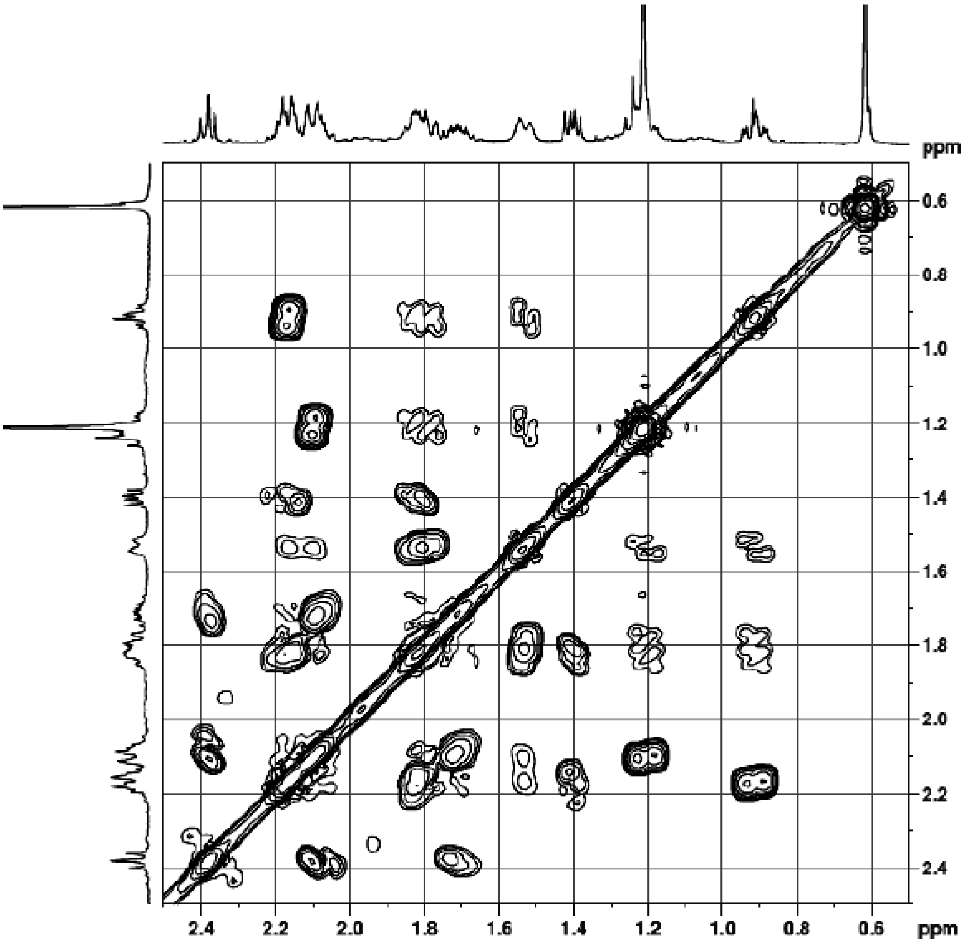

Figure S45. COSY spectrum of **7**

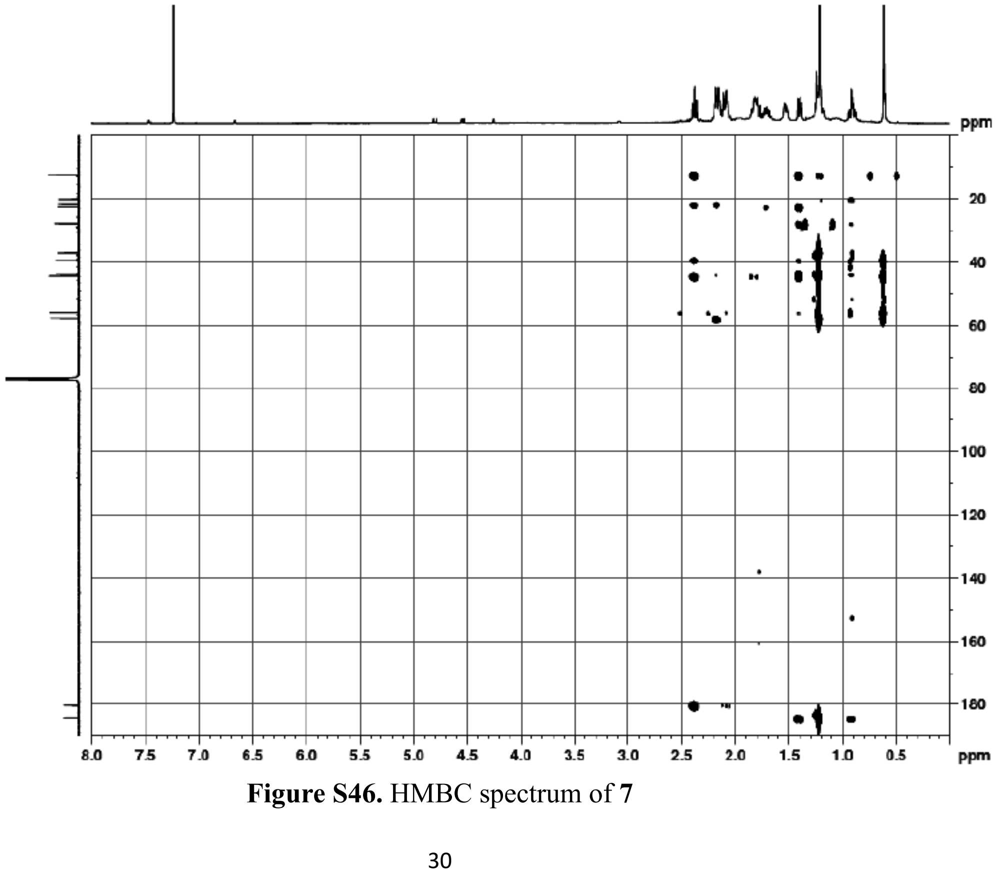

Figure S46. HMBC spectrum of **7**

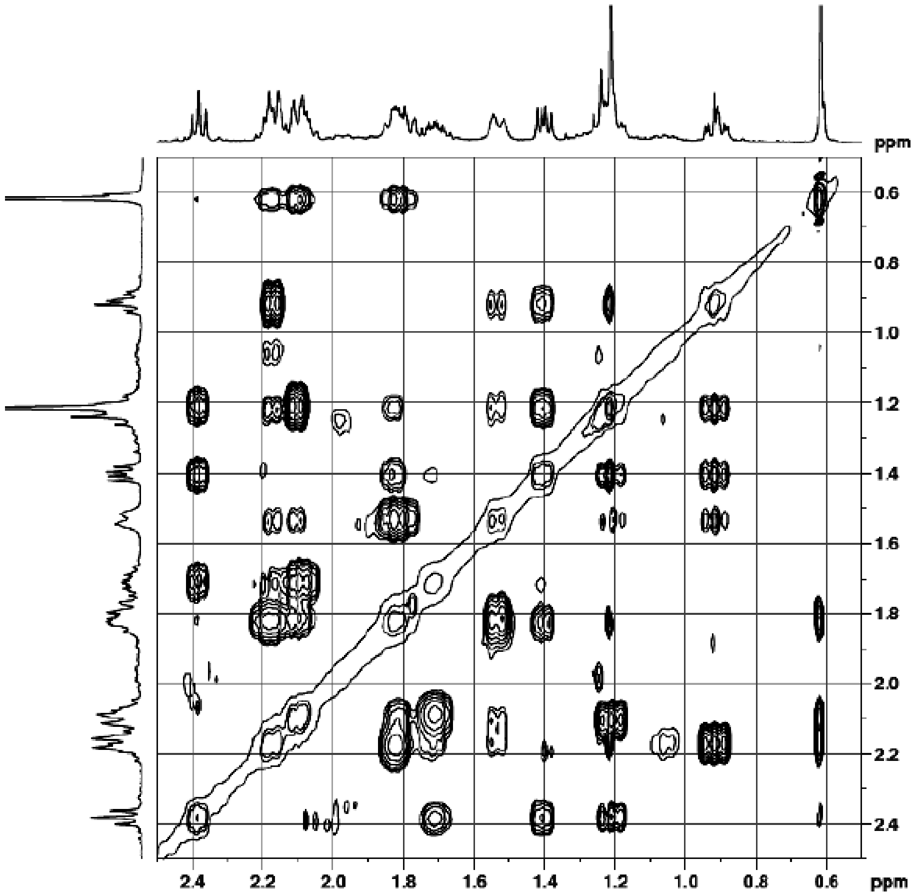

Figure S47. NOESY spectrum of **7**

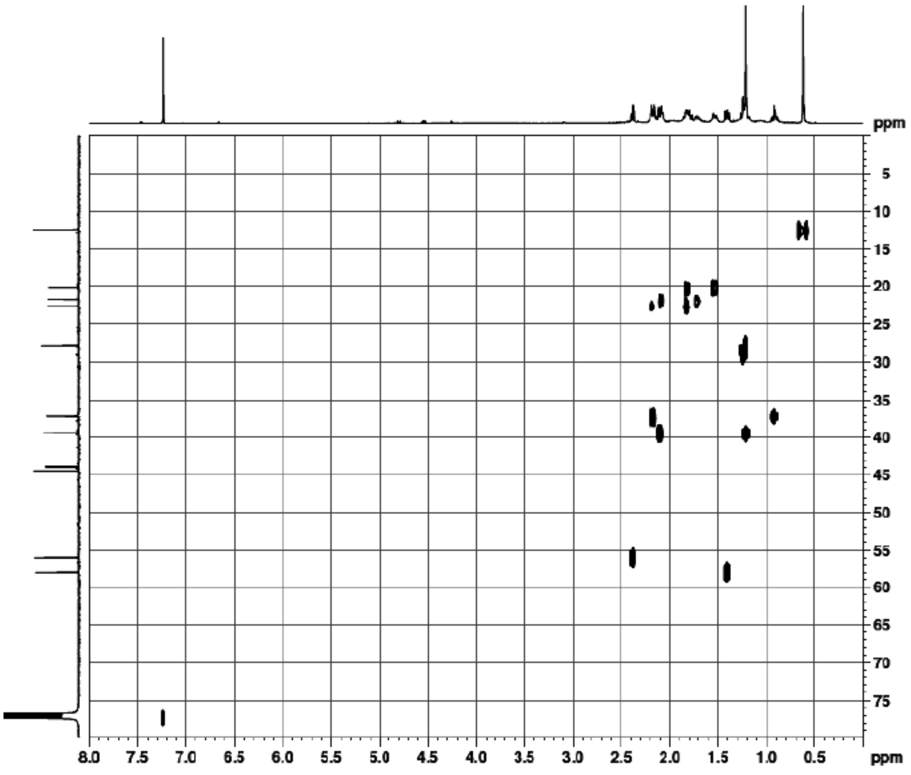

Figure S48. HSQC spectrum of **7**

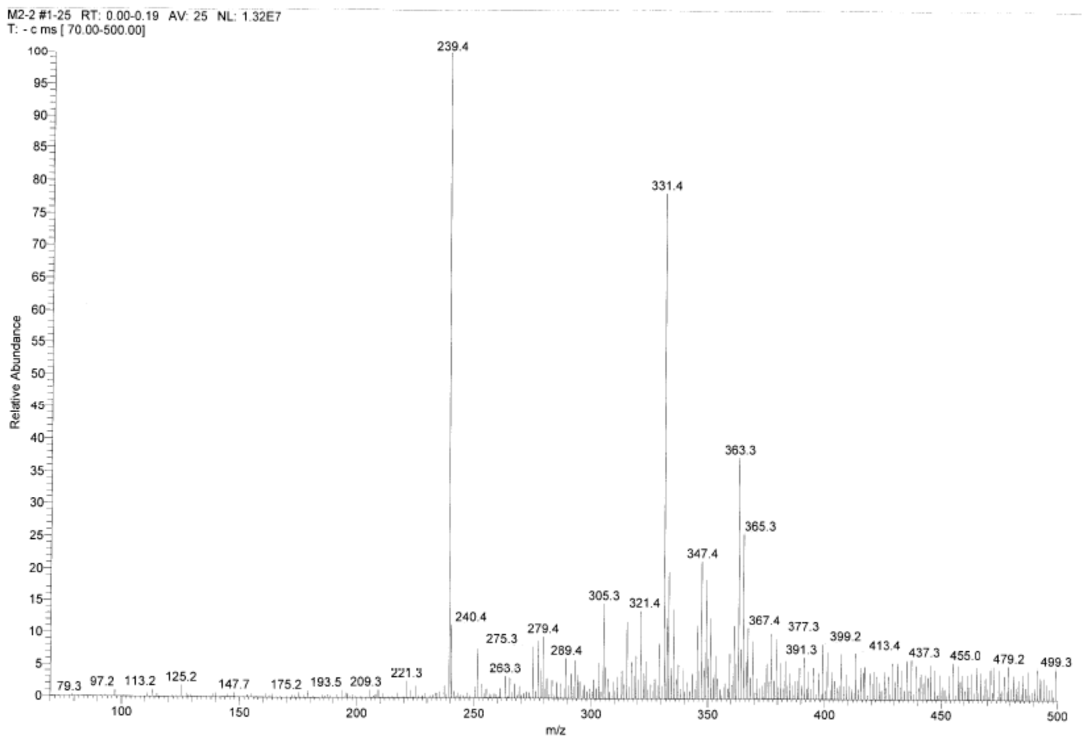

Figure S49. EI-MS spectrum of **7**
